# Towards gender-inclusive job postings: A data-driven comparison of augmented writing technologies

**DOI:** 10.1371/journal.pone.0274312

**Published:** 2022-09-09

**Authors:** Helena Mihaljević, Ivana Müller, Katja Dill, Aysel Yollu-Tok

**Affiliations:** 1 Department of Computer Science, Communication and Business, Hochschule für Technik und Wirtschaft Berlin (HTW), Berlin, Germany; 2 Einstein Center Digital Future (ECDF), Berlin, Germany; 3 Department of Cooperative Studies, Hochschule für Wirtschaft und Recht Berlin (HWR), Berlin, Germany; PLoS ONE, UNITED STATES

## Abstract

Job advertisements are often worded in ways that might pose discrimination risks leading to the exclusion of certain groups of applicants, particularly in relation to their gender. Especially in male-dominated professions or leadership roles, the specific linguistic formulation of job postings acquires relevance if more women are to be attracted to apply. Various technologies have emerged that offer automated text screening, some of them even suggesting alternative formulations to increase gender inclusivity. In this study we analyze four software providers on the German market using a corpus of ∼160, 000 job ads from three different platforms. We identify the relevant social psychological research on gender and language that is at the scientific core of these technologies. We show that, despite sharing a common foundation, the four tools assess the potential for exclusion in job postings in a considerably divergent way on multiple levels of comparison. We discuss the levers in the software pipeline of all four technologies, as well as the potential effect of certain implementation decisions, such as string-based vs. semantic approaches to computational processing of natural language. We argue that the ‘technological translation’ of research is extremely involved and further studies of its use in practice are needed to assess the potential for more gender equality.

## Introduction

The early stages of recruiting are decisive to find viable job candidates [1–3]. Job advertisements, typically provided in digital form, have become one of the main methods to attract new employees during the ‘war for talents’, especially as these are the primary source for assessing job-related information [4]. However, many job advertisements are subject to discrimination risks that might lead to the exclusion of certain groups. A study conducted in Germany in 2018 shows that exclusion risks are found in every fifth job advertisement (*n* = 5, 667), which indicates that the wording and images of many postings do not address underrepresented population groups [5].

One of the most common reasons for exclusion risks in job ads is on behalf of gender [5–7]. Research has demonstrated the presence of gender stereotypes in job postings [8, 9], confirming a congruence of linguistic manifestations of gender stereotypes with gender dominance of the respective positions. Gender stereotypes are generalized assumptions or preconceptions that affect behaviors and judgments which can influence others’ evaluation of work-related skills [10] or career choices [11] but also one’s own self-perception [12–14]. In the context of job applications, gender-stereotypical linguistic constructions can influence how respondents interpret and react to job ads [8, 15–17]. In particular, research indicates that women (in comparison to men) are more willing to apply on behalf of job profiles containing prototypically feminine traits [8, 16–18]. This is consistent with the ‘lack of fit’ model by Heilman [19, 20] by which women, when comparing their own characteristics and competencies with job requirements, might regard occupations described with stereotypically male characteristics less attractive due to the incongruity with self-perception [18, 21, 22].

Given the potential of language choice in job ads to enlarge and diversify the group of applicants, in particular to attract more women to apply, it is hardly surprising that the optimization of job descriptions has opened up a growing business area in the field of HR software. A number of technologies offer automated screening of texts for potential gender-based (and other) exclusions; some even propose concrete replacements to make texts more attractive for women. Often these are marketed with the promise that their use will increase the respective number of job applications. It is therefore important to understand the functioning of such software as a first step in order to assess their impact on reducing gender exclusion in the labor market and the possible implications for gender equality.

In July 2020, we conducted an extensive research of the German-speaking market in search of technologies that address gender-based exclusion through language in job postings. As a result, we found four software providers, one academic institution with an open access tool and three proprietary vendors offering a module within a larger HR software suite. All four promote their approaches with reference to scientific findings in the field of social psychology, especially related to gender stereotypes. Given the volume and broadness of corresponding research on the one hand and the possibilities of technological implementations on the other, the question arises as to what practical challenges emerge when translating social psychological findings into software. Considering that psychological research is often based on small contextualized studies under laboratory conditions, the ambition to automatically evaluate arbitrary job postings with respect to gender exclusion is to be considered very challenging. There is no ‘ground truth’ data set of job ads for which research has measured the gender exclusion potential (under real-life conditions) and which we could use for validation purposes. Therefore, we apply the technologies under study to a corpus of more than 160,000 job advertisements and compare their results along several dimensions. We shed light on the aforementioned practical challenges by focusing on two research questions, which we elaborate in more detail below.

The basis for all four technologies are sets of words for which research in social psychology has demonstrated a (fe)male-attracting or (fe)male-excluding effect. Several empirical studies in this context are utilized by multiple providers, in particular such based on agency and communion, two fundamental dimensions in psychology that play a central role for the understanding of gender stereotypes [23–25]. This raises the question of (Q1) whether the technologies also assess the potential for exclusion in job advertisement texts in a similar way. This is particularly intriguing in light of the fact that, in case of research on agency and communion, “the content and general approach of the [self-developed] lists has varied widely, precluding direct comparisons between studies” [9], which, in addition, span a long period of time and are largely specific to the English language.

A common scientific basis can be translated into software in different ways. Various methods are available to search for specific words in texts, from simple string-based approaches to complex natural language processing (NLP) methods, including machine learning (ML) models. Moreover, the respective technologies go one step further than the extraction and evaluation of individual words. The number of words extracted is combined into an overall rating of the text in question, in the form of a numerical score or a categorical classification such as ‘the text has predominantly male connotations’, or both. This brings up the question of (Q2) what influence the components of such an algorithmic pipeline have on the final result.

We show that the analyzed technologies yield differing evaluations of job ads (Q1), and that each parameter in the algorithmic pipeline has a considerable impact on the final assessment of a text (Q2). This opens up important practice-related questions regarding the development and especially the use of such technologies that we believe should be investigated further in the future, such as: how should HR practitioners decide which technologies to use and to trust, and can such algorithmic decisions be trusted at all given the high sensitivity towards data processing steps? How can developers validate their software and how can scientists from the respective fields contribute to fill this gap?

To the best of our knowledge, our study is the first to systematically assess how current HR software operates to screen gendered wording in job ads. It contributes to the evaluation of automated language-based technologies for personnel assessment and confirms similar findings in the field indicating the need for further validation work to better evaluate and improve current technologies at the intersection of (social psychological) science, language, and human resources (cf. e.g. [26–28]).

The remainder of this paper is structured as follows: we begin with an elaboration of the algorithmic levers in the implementation of considered software products and present the main scientific theories and studies on gender-specific word lists utilized by the providers. The subsequent section compiles key information about the technologies examined. Then, results of a comprehensive empirical analysis based on a corpus of real-world job ads are presented, comparing the technologies statistically at the level of derived gender categories, the scores underlying them, and the gender-specific vocabularies. The technologies are compared both on the basis of individual texts and on the basis of groups of job ads that represent different segments in the labor market. After a summary, the implications of our study for practice are derived, and questions for future research are formulated.

For the rest of the text, it should be emphasized that we do not understand the terms ‘woman’ and ‘man’ or ‘feminine’ and ‘masculine’ as monolithic categories, but rather as processualities that are formed through social attribution processes. We stand for an ambiguity and deconstruction of the terms as to include gender fluidity; however we were confronted with the dichotomous implementation of the studies referred to in the following. This reference is intended to encourage breaking down heteronormative gendering and operationalizing it as a non-binary factor.

## From word lists to software

**Fig 1:**
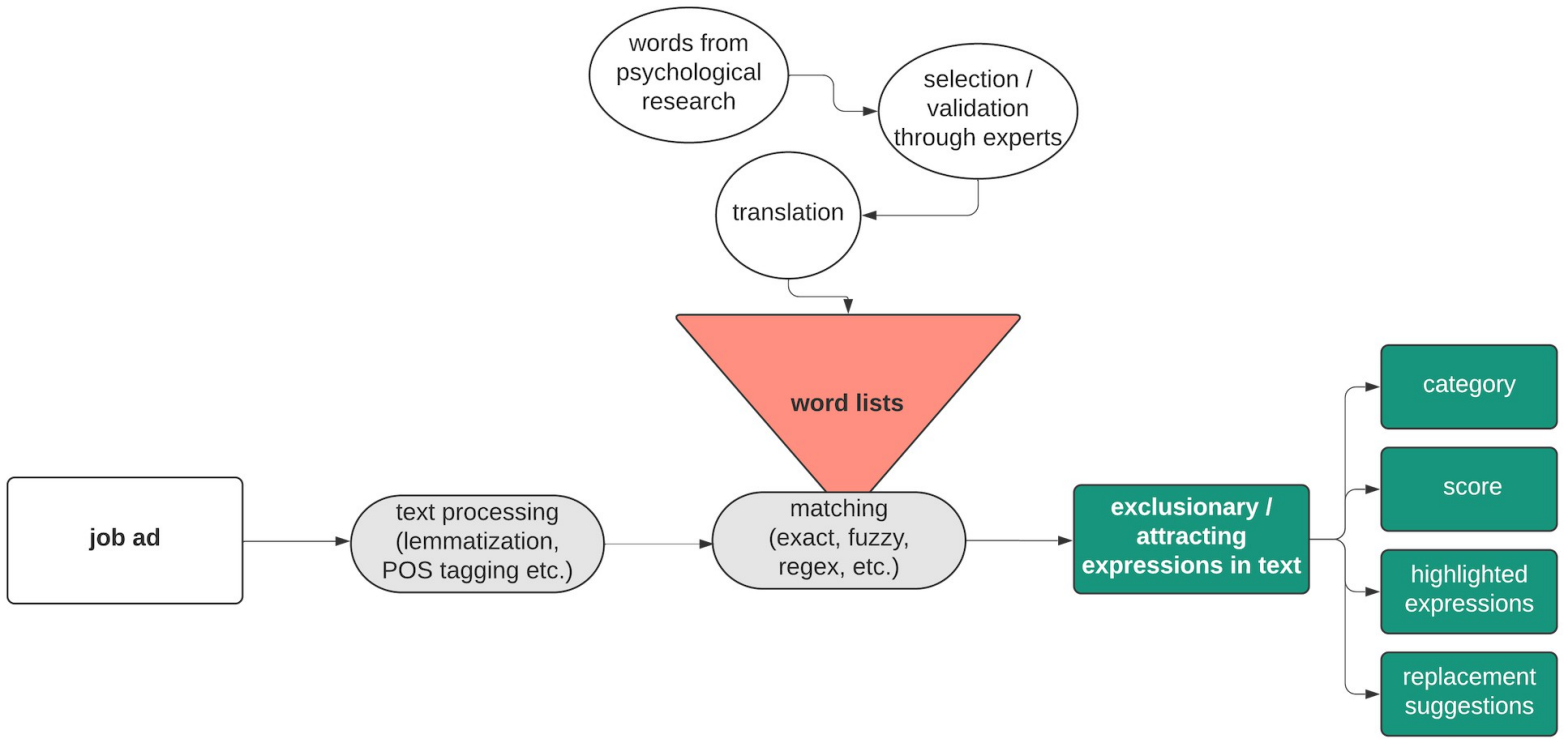
Schematic description of a pipeline to build augmented writing software to detect and correct gender-based exclusion based on research on gender stereotypes.

As mentioned in the introduction, all four technologies examined in this paper work with lists of words or phrases that have been shown to have a female-attractive or female-exclusionary effect in scientific studies—in what follows we refer to such a list of expressions as a dictionary. Before we elaborate on the respective scientific results, we will roughly sketch typical steps that a technology would need to implement in one way or another (see Fig 1). We will show later that all these adjustment levers have an effect on the final evaluation of a text.

### Schematic representation of the software pipeline

A dictionary can take different forms: it can consist of single words such as ‘leadership’ or ‘emphatic’; it could include phrases such as ‘independent working style’; it can include word fragments such as ‘indiv’ that stands for all words starting with said prefix. Furthermore, additional information is conceivable, e.g. to enable disambiguation of a particular expression, as for the word ‘kind’ (‘type’ or ‘caring’). Here, procedures like part-of-speech (POS) tagging can help to distinguish meaning based on grammatical function of the word, such as noun vs. adjective. Context information, as represented by word embeddings, for instance, can be used for further disambiguation, as exemplified by the word ‘mean’ that could refer to ‘average’ as in average salary or to ‘not nice’. For background on NLP see e.g. [29, 30].

The text to be evaluated must thus be processed to allow matching with a dictionary. Typically, the text goes through the same preprocessing as the sources from which the dictionary was extracted. More specifically, if our list consists of lemmata (a particular canonical form of a word), the text should also be lemmatized. If the dictionary contains additional information such as POS tags, these can only be made usable if POS tagging is also performed for the text in question. For the matching itself various possibilities exist such as exact or fuzzy matching of token or lemmata (and the respective logics can also be already partially covered by the steps described before). The found matches can then be employed in different ways: for instance displaying them to users, possibly with an additional ranking or weighting. Furthermore, an overall conclusion can be extracted as a categorical statement such as ‘the text has a tendency to exclude women’ or as a numerical figure. The latter could be estimated as the proportion of exclusionary terms in the entire text or some function thereof.

Moreover, words and phrases can be represented as multidimensional vectors using embeddings that encode semantic information to some extent, in the sense that vectors of words that occur frequently in similar contexts are close to each other. This can be used to generate suggestions for replacements: assume, for example, that the word ‘strengthen’ is associated with an exclusionary effect; then, one could suggest to replace it by a close word in the embedding space (thus with similar meaning), such as ‘empower’ that is associated with a positive effect.

### Word lists stemming from social psychological research

From the perspective of technological utilization, three strands of psychological research seem to have been instrumental in shaping the understanding of how advertisement texts might discourage women from applying: research on the textual representation of agentic and communal traits, language analysis through the Linguistic Enquiry and Word Count (LIWC) software focusing on the differences between women and men especially in the use of function words, and the representation of personal characteristics in terms of traits vs. behavior.

At this point it is important to mention the immense body of research on the relationship between gender and language: how men and women are described by others, what characteristics they are associated with, how they describe themselves, or differences in the way they use language. A variety of differences in linguistic focus exists from analyzing specific words or phrases, linguistic diversity, use of questions and negations, or grammatical constructions. Next, we will mainly focus on those research studies that have been instrumental in the creation of gender-associated dictionaries used by the technology providers analyzed here. For thorough elaborations we refer to research in the respective fields (see e.g. [8, 9, 31]).

#### Word lists reflecting agentic and communal traits

The constructs of agency and communion go back to Bakan [32] and reflect the idea that there are two fundamental themes in human life: the pursuit of individuality or realization of one’s goals (agency) and the pursuit of social relationships (communion) [33]. Also known as the Big Two [34, 35], they are widely agreed to represent fundamental dimensions in the perception of the self and others [23, 36–40].

Despite the lack of a universally accepted definition, agency and communion have been operationalized in numerous studies, including those relating personality traits and gender. As part of this, agency is linked to men who are considered to be more dominant, competitive, and assertive, while women are rather categorized in the communal dimension, as they are perceived to be more unselfish, emotional and empathic [24, 25, 41, 42]. In the last two decades, a multitude of studies have examined the presence of gender stereotypes in job advertisements on behalf of agentic and communal wording, showing that e.g. postings of male-dominated jobs contain more agentic than communal words comparing to female-dominated job ads [9] or that “masculine wording reflected in real job advertisements primarily serves to keep women out of the areas that men typically occupy” [8]. In this regard, the work of Gaucher et al. [8] represents a landmark and recurring reference point in academic research as well as in the technologies examined in our study. From the perspective of technological utilization, this particular research plays a pivotal role for two reasons: For the first time, agentic and communal traits were transformed into lists of words and word stems, consisting of 42 ‘masculine’ and 40 ‘feminine’ words, prepared to capture agency and communion in arbitrary natural language texts. While previous research had already described these traits [43–47] such as being competitive or supportive, the authors went a decisive step further and transformed these trait descriptions into searchable words. The second reason for the importance of this work lies in a multi-stage examination of job advertisements: the authors proved “subtle but systematic wording differences within a randomly sampled set of job advertisements”, showing that job postings in male-dominated fields used more masculine wording while no difference could be shown in the presence of feminine formulations [8]. The effects of wording, e.g. on job appeal, personal skill, and belongingness, has been tested in experimental studies. One of the main conclusions is that job ads for roles more strongly associated with men, such as in the IT industry, also contain more masculine (i.e., agentic) wording, which women feel less addressed by. The gender-reversed effect, on the other hand, was not observed in most studies [8, 16, 17, 48].

#### Utilization of the LIWC

Researchers have used manually compiled dictionaries such as those of Gaucher et al. [8] to study agency and communion in natural language. As pointed out by Pietraszkiewicz et al. [9], both the content and the methodological use of the dictionaries varies in the respective studies, making comparability of the obtained results enormously difficult. Especially the question of how to match a text with lists of words and word fragments and to count and weight the hits found is not covered by corresponding research. It is important to bear in mind that most of the work prior to Gaucher et al. [8] did not aim to process arbitrary and large amounts of texts in search of findings on agency and communion. Rather, in experimental studies, texts were deliberately manipulated and the effect of those linguistic changes on probands was measured. The step from targeted text manipulation by humans to automated search for words in arbitrary corpora may generally seem straightforward; however, this is one of the typical fallacies in the context of computer-assisted text processing. As we will show in the following chapter, different implementations can yield significantly diverging results.

Gaucher et al. [8] did not count words in the collected job ads by hand; instead they processed them using LIWC2007, a software for language analysis that allows to find groups of words and compute their ‘density’ within an arbitrary text. LIWC was developed by researchers around James Pennebaker, releasing a first version in 1993, “to provide an efficient and effective method for studying the various emotional, cognitive, and structural components present in individuals’ verbal and written speech samples” [49]. For this purpose, words were collected from various sources, such as emotion questionnaires or thesauri, and assigned to the respective categories after being rated by several independent experts [31]. The latest version consists of a dictionary of >6,000 words and word stems that correspond to one or more categories. For instance, the word ‘cries’ is part of (at least) the categories ‘sadness’, ‘negative emotion’ or ‘verbs’, thus its occurrence in the target text would yield an increment for each of these subdictionary scale scores [49]. The categories and subcategories span a broad range of concepts, from grammatical constructs such as pronouns and articles to psychological processes such as positive emotions or anger.

As pointed out by Pietraszkiewicz et al. [9], agency and communion are not directly represented as LIWC dictionaries. It is possible to embed custom dictionaries within LIWC, such as those by Gaucher et al. [8] but other studies have also investigated agency and communion indirectly by using LIWC dictionaries such as ‘Money’ and ‘Achievement’ or ‘Family’ and ‘Social processes’, respectively, as proxies (see e.g. [50]). It is also worth mentioning that Pietraszkiewicz et al. [9] has applied LIWC to build dictionaries that are considered psychologically relevant to agency and communion based on expert evaluation, yielding 192 words for agency and 184 for communion. However, even independently of such adaptations, the LIWC can and is used directly for linguistic analyses of gender-based differences. At the heart of the idea behind the LIWC is the role of so-called function words: words that do not represent content such as verbs, nouns, or adjectives but “are used to ‘glue’ other words together” [51]. In the 1980s, Pennebaker and colleagues first examined letters from depressed patients and showed the power of function words to predict depressive illness [52]. This initial finding was followed by others studying the question of how language is related to personality. In their meta-study, Newman et al. [51]. investigated differences in the way women and men use language based on 14,000 texts, thus providing a stronger empirical basis that managed to replicate some of the earlier findings but not all and yielding explicit dictionaries related to sex-based differences in word choices. Given the role of language, these differences “are likely to play a central role in the maintenance of gender stereotypes” [51], and can thus be utilized as part of augmented writing technologies for exclusion detection.

#### Representation of personal characteristics using traits vs. behavior

The third line of research that has been incorporated into the technologies under study stems from the Linguistic Category Model (LCM) [53, 54], a tool for language analysis characterized by its classification of predicates in terms of abstractness. One of the major findings that allows to use LCM to study gender-based effects is that “if personal characteristics are presented as traits, […] using nouns and adjectives, people tend to consider this information as more revealing about a person’s nature than if the same information is presented in terms of behavior”, using verbs [16], cf. [55]. This suggests that if desired characteristics of potential job applicants are formulated as traits, they are seen as something that one either has or doesn’t. This increases the risk that recruiters might base their own assessment on stereotypes about women’s presumed nature [17], whereas presenting the requirements as an action or activity shifts the focus to the motivation to perform the corresponding task rather than to the underlying dispositions [16]. Thus, women might be more inclined to apply for a job associated with prototypically masculine characteristics if the requirements are formulated in terms of behavior instead of traits, and vice versa. Experimental studies with students support this theory at least for women [16, 17] suggesting that “organizations may increase the number of women applying for particular jobs by changing the presentation form of the advertisement” [16].

### Adaptation to the German language

The cited social psychological studies were conducted predominantly in English, thus the corresponding findings, particularly the word lists, need to be transferred to German. The words associated with agentic and communal traits were typically translated directly [56, 57], with appropriate adaptation for word stems and fragments. For the LIWC, a partial German-language version was created in 2003; its quality and validity were tested by Wolf et al. [58], showing a good equivalence of the German version with the English original for the majority of the LIWC categories. For some of them, however, substantial differences were observed and further research was recommended. With the LIWC2015 release, the German dictionaries and categories were fundamentally revised.

Another peculiarity of the German language in this context is the fact that most job titles have a feminine and masculine form, the latter often being used as the generic form for all genders. Research has shown that humans associate the generic masculine predominantly with men, while the use of both word forms can weaken this so-called male bias in mental representations [59]. If an organization advertises a position covering both masculine and feminine terms for job titles, both women and men rate the position and organization as more attractive and are more likely to apply [60]. At the same time, there is less preference for men by recruiters and women are more likely to be hired for management positions with a similar probability as men [57] Yet, the usage of both forms is rather rare and many employers add the notion ‘m/w/d’ to the generic masculine job title in order to (formally) include other genders. One of the main reasons behind this notion is the General Equal Treatment Act (AGG), according to which employers may not discriminate against applicants on the basis of gender or sexual identity; the third option ‘d’ stands for ‘diverse’ and refers to intersex or non-binary gender identities.

## Technologies for detection of gender biases in job postings

Below we compile key information about the technologies examined, based on information made available by the providers on their websites, in publications and in personal communications. A summary is displayed in Table 1.

**Table 1:** Overview of the four technologies for detecting gender-exclusionary language in German-language job postings.

	FührMINT	BetterAds	100W	Stafff
**operator**	TU München	milch & zucker AG	100Worte Sprachanalyse GmbH	Visualino AG
**free access**	yes	no	no	yes
**available since**	2019	2020	2017	2019
**dictionaries public**	yes	no	no	no
**displays matched words**	yes	yes	yes	yes
**displays gender category**	yes	no	yes	yes
**gender score**	no score computed	exact score displayed	score indicated on a colored slider	score indicated on a colored slider
**provides explanations / suggestions for replacement**	no	yes	yes	no
**underlying psychological theories**	agentic / communal traits	agentic / communal traits, LCM	agentic / communal traits, LIWC	agentic / communal traits

### FührMINT

The FührMINT Gender Decoder (hereinafter referred to as FührMINT) is a web application of the Chair of Research and Science Management at the Technical University of Munich. It was developed within the scope of the eponymous research project FührMINT which “investigates success factors for women in academic STEM fields” [61]. The application features a web form in which a job advertisement text can be inserted. The software was created with the aim to develop a German-language version of the English-language Gender Decoder by Kat Matfield [62].

#### Underlying theory and dictionaries

**Fig 2:**
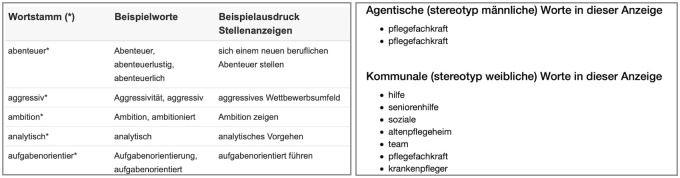
Word stems and examples of matching words in FührMINT. Left: Sample of agentic word stems with word and context examples in job ad texts. Right: Example of extracted agentic and communal words. The word ‘pflegefachkraft’ (‘nursing professional’) matches the agentic stem ‘kraft’ and the communal stem ‘pflege’, and is displayed and counted w.r.t. to its occurrence in the text.

The tool seems to be based solely on agentic and communal traits; the main scientific publications, including the study by Gaucher et al. [8] as the central scientific source, are listed on the project website. The underlying dictionary features 61 agentic and 63 communal word stems that are listed on the website together with examples of matching words and their usage in job advertisements (see Fig 2). We could not find any information on the website about how English-language dictionaries were translated into German. However, among the literature references are studies that also had to rely on translations into German, and it is plausible that these translations were used as much as possible. The aforementioned studies themselves state that “words were translated and back translated from English to German by independent bilinguals” and that tests were conducted to ensure that the stereotypical perception of the terms was preserved.

#### Computation of scores and categories

The software extracts words that are considered agentic or communal and displays them in separate lists. If the total number of communal words predominates, the text is assessed as ‘predominantly communal (stereotypically female) in wording’, and analogously when agentic words dominate. If there are equal numbers of terms from both groups, the wording is considered neutral. In addition to the assessment, a brief explanation is provided.

#### Text processing and matching

We could not find any technical description of the text processing pipeline and word-match counting scheme used in the software. However, project members kindly provided us with their code from which it can be seen that a simple pipeline is implemented: after replacing certain special characters by spaces, the text is tokenized and words that complete a stem from either of the two dictionaries are extracted and counted. (The providers recommend some text cleaning such as removing hyphens from the text before using the tool). A token can thus be considered both agentic and communal despite the fact that both lists of word stems are disjoint, as shown in Fig 2 with the word ‘Pflegefachkraft’ (‘nursing professional’). In fact, words like ‘Pflegehilfskraft’ (‘nursing assistant’) match with more than two stems so that this token is counted three times, although it appears only once. Furthermore, the tool lists all word occurrences instead of the unique matches; thus, a text that contains multiple repetitions of a single communal word like ‘cooperation’ would be considered communal overall, despite the presence of almost as many different agentic words.

### BetterAds

The company milch & zucker offers a range of recruiting and talent relation management products as part of the Beesite HR Suite. One of them is Beesite BetterAds, an Augmented Writing tool that is marketed as using artificial intelligence (AI) to enable recruiters to write better job posting texts. The company promotes the technology in a white paper as follows: “On a scientific basis, you can optimize the texts of your job ads quickly and easily. For more reach and up to 75% more clicks on the apply button. And that’s just by tweaking a few phrases” [63]. We could not find any further information on corresponding studies that would confirm the marketing promise; however, the company has a job board called Jobstairs in its portfolio, which could be used by the company to test the effect of their algorithms.

#### Underlying theory and dictionaries

**Fig 3:**
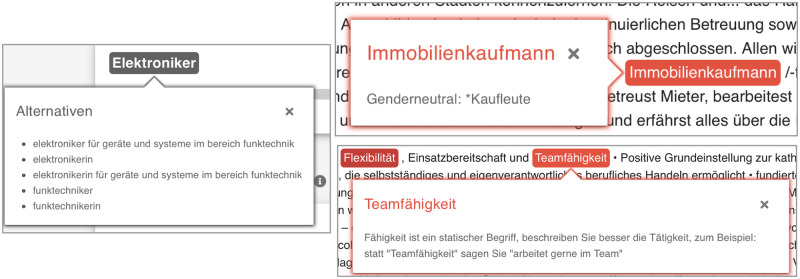
Examples of words highlighted in BetterAds. Left: word alternatives for the job title ‘Elektroniker’ (‘electronics technician’) in the masculine base form include suggestions for more detailed specification of the job title as well as the feminine base form ‘Elektronikerin’. Use of word embeddings can be seen, for example, in semantically similar suggestions such as ‘Funktechniker’ (‘radio technician’). Top right: The word ‘Immobilienkaufmann’ (‘real estate agent’) contains the suffix ‘Kaufmann’ (‘merchant’, masculine base form containing the word ‘Mann’, meaning ‘man’), which is suggested to be replaced by the gender-neutral phrase ‘Kaufleute’. Bottom right: Utilizing LCM theory, the tool suggests replacing the static term ‘Teamfähigkeit’ (‘team capability’) with a description of the activity such as ‘likes to work in a team’.

The tool’s scientific basis is explained in the preliminary study [64], in which the company collaborated with academics from the field of work and organizational psychology. The psychological foundations can be assigned primarily to the Big Two theory and work derived from LCM. An application of the latter can be seen in Fig 3 where the tool suggests replacing the noun ‘Teamfähigkeit’ (‘teamwork skill’) by the verb phrase ‘arbeitet gern im Team’ (‘likes to work in a team’). The dictionaries, however, are not public.

BetterAds draws on both English- and German-language literature on gender stereotypes, translating the English-language dictionaries used. In [64] the authors justify the validity of the transfer with reference to research according to which “there are more similarities between Western countries in gender stereotypes than differences”.

The company refrains from gender-connoted naming of the respective word categories and refers to them instead as pull and push words. Their choice is primarily justified by the fact that scientific findings show that the one group of words (push words) has a negative effect on all applicants, albeit on women more strongly than on men, and thus the avoidance of corresponding terms also makes sense beyond the desire to motivate more women to apply.

It should be further noted that the tool checks for the use of gender-fair language. As can be seen in Fig 3, it proposes replacing the generic masculine in the titles of roles such as ‘Elektroniker’ (‘electronics technician’) with inclusive or gender neutral variants.

#### Computation of scores and categories

BetterAds calculates a performance score using mainly the number of push and pull words. For its calculation, words of both lists are weighted once or twice depending on the strength of the respective scientific evidence. According to Böhm et al. [64] the score is calculated as follows: The sum of the weights of the push words is subtracted from that of the pull words and divided by the number of all words found, counting the words with weight 2 twice. The final performance score equals the value of the sigmoid function applied to the previously calculated linear combination and is thus a number between 0 and 1, where the value 0.5 corresponds exactly to having equal numbers of push and pull words, counted with weights. A score larger than 0.5 means that there are more pull than push words and vice versa. In our empirical study, we found that the score deviates from this formula. According to the most recent version of the company’s description of their tool, the overall gender sentiment is also taken into account in the calculation [63].

Unlike the three other technologies, BetterAds does not explicitly assign a text to a category. In the GUI, the score is displayed along with a traffic light donut chart colored in red, orange or green, depending on whether the score is below 0.33, between 0.33 and 0.66, or above 0.66. Push words are highlighted in red and pull words in green. A darker shade indicates that the word is weighted twice. Furthermore, the tool shows explanations for each highlight along with suggestions on how the encountered push word could be replaced. During our experiments, we found that BetterAds also classifies occasional words as both push and pull words. However, this conflict is resolved in the GUI, as each highlighted word has a unique color.

#### Text processing and matching

From both the company’s self-representation and our tests, it is clear that BetterAds uses a more complex pipeline of algorithms, including the computation of word embeddings and projections on selected axes in the corresponding vector space.

### 100W

100W offers a range of products in the field of so-called Psychological AI. The company claims that “based on their words, 100W gives insight into people’s personalities, relationships, feelings, ways of thinking and needs. In combination with consciously formulated words, our tools make the unconscious transparent in communication” [65]. Their overall approach combines social psychological theories, in particular works of Pennebaker and collaborators according to which the frequency with which we use certain words makes statements about our personality, with state-of.the-art NLP methods.

The alleged scientific nature of the technology is brought to the fore. The website states, “The foundation of 100W Psychological AI is a worldwide recognized scientific method. We meet the highest standards regarding validity.” [65] The company provides several white papers outlining their approaches. Their products address communication aspects in various domains, including HR. One of the functionalities of the technology is the check for gender bias in job advertisement texts.

#### Underlying theory and dictionaries

The idea and approach behind this functionality are described by Burel et al. [66]: Inspired mainly by Gaucher et al. [8], the company wanted to check if their results could be replicated using a dataset of job postings from the German market. Based on social psychological literature, in particular the theory on agentic and communal traits and on reviews by human expert annotators, they formed dictionaries containing about 1,300 words, which in turn were integrated into the entire suite via their own ML-based language processing pipeline. The dictionaries are not public; the mentioned study states that parts of the dictionaries can be found in the appendix but we could not identify such an appendix. It is worth noting that the characteristics usually associated with masculinity or femininity from the LIWC do not appear to be used here, resorting instead to [8]as well as to further relevant literature in this direction.

100W do not explicitly write about the transfer of English-language literature into German. However, they do state that research literature is in general manually evaluated by annotators as part of an internal review process, and it seems plausible that the necessary translation work is part of this process. Moreover, since the LIWC plays a significant role for their software, we assume that the German translation is utilized as far as possible.

#### Computation of scores and categories

**Fig 4:**
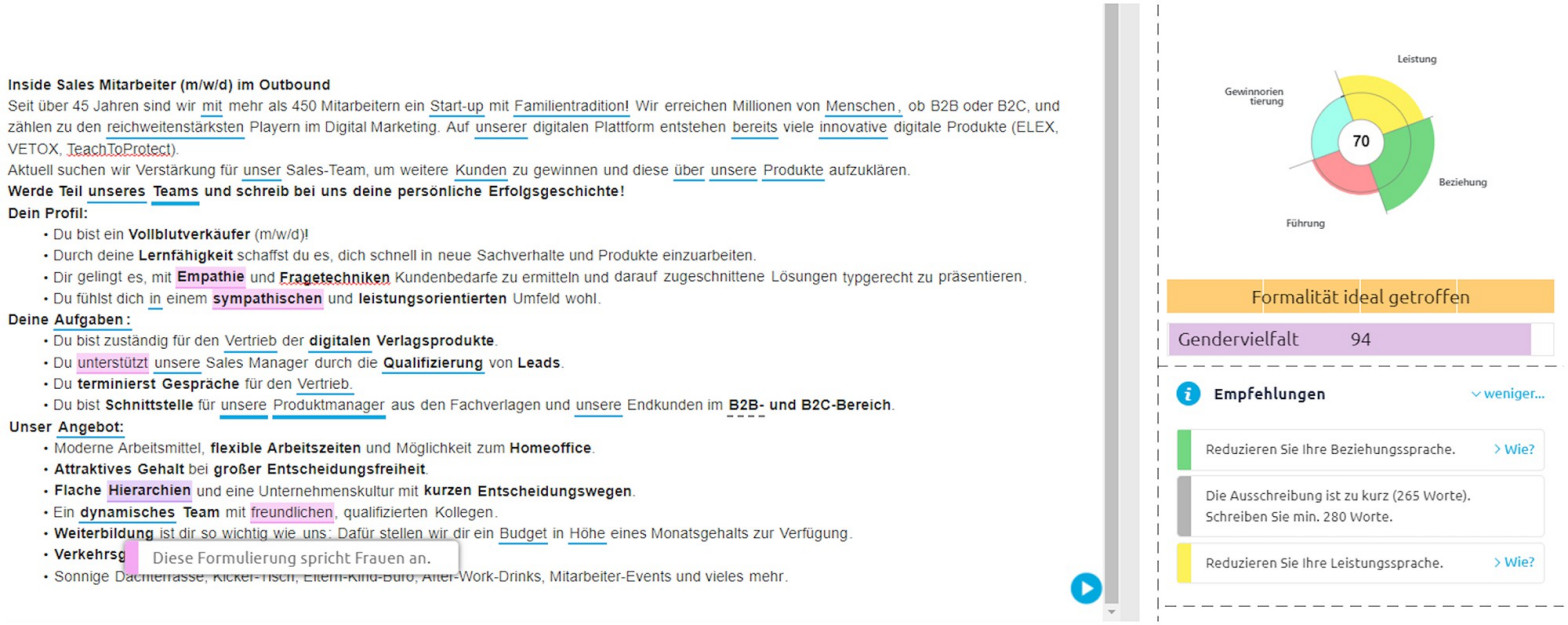
Graphical representation of a text analysis using the tool from 100W. A gender score slider is shown on the right. Words corresponding to different psychological profiles are highlighted in different colors [67].

In the GUI, displayed in Fig 4, words associated with certain motifs are highlighted. A mouse-over text provides short explanations. Words and phrases associated with masculinity and femininity are color-highlighted; a ‘gender score’ is calculated for each text and displayed only as a relative value on a color slider that ranges from ‘feminine’ to ‘masculine language’. The presentation of the Gender Score Slider changed during the course of our project; Fig 4 shows the current variant from the provider’s website, in which the slider is titled ‘Gender diversity’ without making clear how exactly the number and color are to be interpreted and whether this is meant to suggest an approach beyond gender binary (which, however, cannot be part of the functionality). An overall categorical classification of the text is also provided, for example ‘Promote diversity—use the following words for more diversity’.

#### Text processing and matching

According to the company’s website and reports, POS tagging and word embeddings are mainly used to to disambiguate word meanings and to ensure that the extracted words also have the desired meaning.

### Stafff

**Fig 5:**
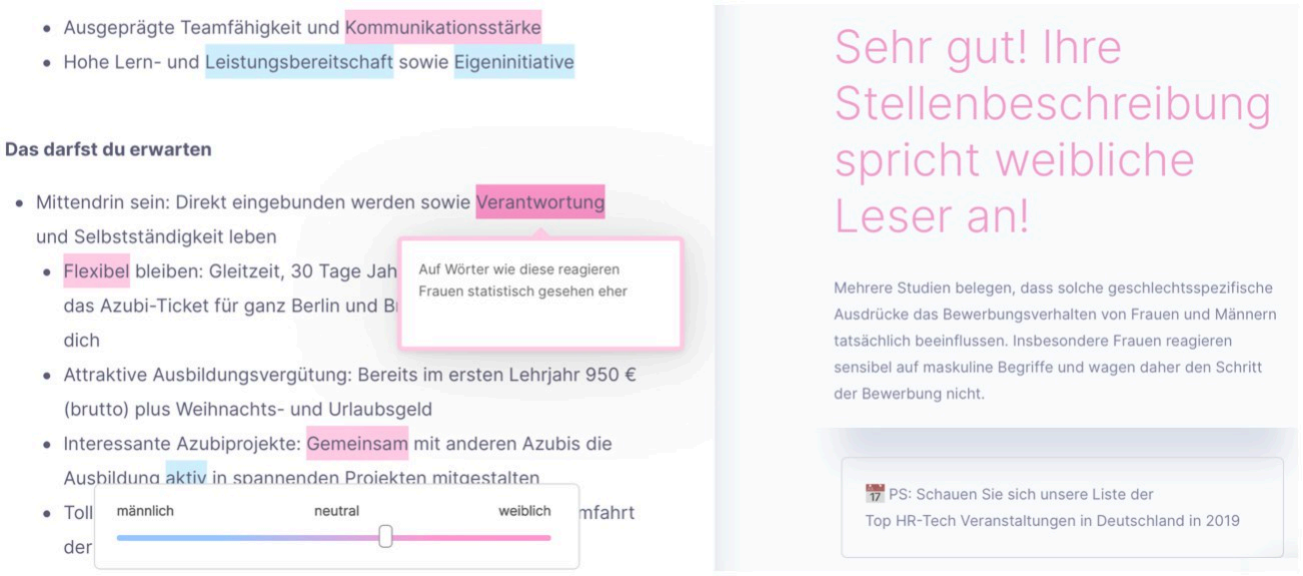
Screenshot of Stafff’s frontend. Feminine words are highlighted in pink, masculine in blue. The slider indicates the gender score for the entire text, and a short text declares that studies show that women are particularly sensitive to masculine terms.

Stafff was a startup from Hamburg-based Visualino UG, which offered an HR software suite described as data- and AI-based. As of March 2019, their product provided gender code checking of job advertisements as a freely available web application, among other functionalities. As of December 2020, the Stafff software suite is no longer available. Upon request, the company said that the gender bias checker will be integrated with other technologies from the operator, which is why we decided to further consider their product as part of our analysis. The software highlighted words and phrases in text that it considered to have masculine or feminine connotations. A ‘gender bias’ slider ranging from male (colored in blue) through neutral to female (colored in pink) indicates how balanced the text is, as assessed by the tool, and which gender is more likely to be addressed, without showing a specific numerical score (see Fig 5).

#### Underlying theory and dictionaries

The program claims to be based on several empirical studies on the topic of word selection in personnel recruitment. The exact studies are not explicitly mentioned, except for [56], which is based on the lack of fit theory and agentic and communal traits. The lists of words associated with one of the genders were not listed on their website. The referenced study, that was also used by FührMINT, itself used English-language dictionaries that were translated independently, so it can be assumed that Stafff relied significantly on these translations.

#### Computation of scores and categories

The company did not reveal any details about the inner workings of their software, including the calculation of the score underlying the slider. However, by inspecting the Java Script code of the web form we were able to partly reconstruct the score computation: the score is a number between 0 (maximally appealing to men) and 100 (maximally appealing to women), with the value 50 representing neutrality. Each matched expression contributes a value of 5 in the respective direction within the given boundaries, counted with multiplicity according to the number of rules that matched the expression. Thus the difference between the absolute numbers of feminine and masculine expressions solely determines the score. A text scoring above 50 (i.e. the total number of feminine expressions predominates) is assessed as ‘appealing to female readers’ and vice versa.

#### Text processing and matching

Again, using the Java Script code of the web form, we were able to determine that the software searches for certain terms using regular expressions which are generally difficult to interpret. As with FührMINT, an expression can be matched by several rules, which in rare cases can classify the term as both feminine and masculine. It should be noted that numerous aspects have an impact on the overall number of matches in Stafff’s tool, from case sensitivity to the number of empty lines between expressions, which is presumably of little surprise to those who have ever programmed with regular expressions.

## Empirical analysis of the technological approaches

To find out how the four technologies evaluate real job advertisements on the German-speaking market, we crawled between October 19th and December 16th 2020 job ads from three job platforms: the job portal of the German Federal Employment Agency (FEA) as a generalist and public platform; Ausbildung.de that offers apprenticeships as a specialized and private job board; and Google Jobs as a meta search engine for job vacancies.

For each of the services we have crawled as much data as possible within the limitations of the respective providers. To do so, we utilized filters provided by the platforms: In the case of FEA we combined the offer type (job or training), the working time model (part-time or full-time), and the time limit (temporary or permanent). Within each filter combination, the data was sorted by timeliness and all available job ads were stored. Additionally to the Germany-wide sample, we collected a second sample consisting of job postings limited to the Berlin area. Data from Google Jobs was crawled using Serpapi’s GoogleClient, providing combinations of German federal states and available job categories (e.g. GC01 for ‘accounting and finance’) for filtering. Data from Ausbildung.de could be retrieved in full.

BetterAds and 100W offered us access to their respective APIs to analyze the corpus data; for FührMINT we reimplemented the code logic using the available word lists; however, at a later date, we gained access to FührMINT’s code and were able to confirm that our implementation followed the same logic. We sent automated HTTP requests through Stafff’s web form.

Our collection and analysis methods complied with the terms and conditions of the respective data sources.

For the analysis, we used the full job postings without the titles. Restricting the analysis to dedicated text passages would not have been possible due to a lack of reliable and consistent information on text zones such as company description, job description, or required hard skills and personality. However, it makes sense to look at the texts as a whole, because the technologies themselves do not automatically zone the texts, and because it can be assumed that users would also apply the tools to an entire job ad.

The originally crawled data was not preprocessed before being analyzed through the considered technologies. Therefore, our corpus also contained misspelled words (e.g. compounds of consecutive words due to missing space character), in particular in texts from Google Jobs, some of which were also among the expressions extracted by the tools.

After postprocessing and validating the crawled data, we have kept a total of *n* = 160, 246 job postings, as shown in Table 2.

**Table 2:** Overview of the corpus, where ‘word’ refers to a token created by splitting at white space and selected special characters.

	FEA	Google Jobs	Ausbildung.de	Total
**number documents**	76,963	74,114	9,169	160,246
**word count: median**	201	229	218	214
**word count: mean**	213.47	239.28	239.83	226.92
**word count: standard deviation**	105.75	136.31	123.90	122.52

The technologies use different labels for the two groups of extracted words and the resulting categorical classification. For example, FührMINT refers to the terms as ‘communal’ and ‘agentic’, respectively, while BetterAds has replaced a gender-related way of speaking with the terms ‘pull’ and ‘push’. 100W and Stafff both use the words ‘feminine’ and ‘masculine’. For uniformity, we use the terms ‘female-connoted’ and ‘male-connoted’ in the following analyses when talking about the categories of the extracted words.

We assessed the technologies based on the corpus, both individually and in comparison with each other. In particular, we compared the gender categories, the scores, and the extracted female-connoted or male-connoted expressions. It should be noted that job postings from Google Jobs were not analyzed through Stafff’s tool due to the fact that this tool was taken off the market in the middle of our analysis phase. Thus, we compare the technologies based on two samples, one including Google Jobs but excluding Stafff (*n* = 160, 246), and another based on data without Google Jobs (*n* = 86, 132) but using all four technologies. The base sample is named in each case.

### Gender categories

As described in the previous chapter, BetterAds does not explicitly assign a text to a gender category. We therefore followed Böhm et al. [64], in which the score 0.5 is suggested to be gender neutral, and the values below or above are associated with the corresponding categories. Recall that the real calculation of the score is slightly different, so the texts with score 0.5 do not (necessarily) coincide with those texts that have the same number of push as pull words.

As can be seen in Table 3, the proportions of the gender categories differ enormously between the respective technologies. FührMINT categorizes the majority of texts as female-connoted (72%), while BetterAds and 100W have an opposite distribution with 80% of texts categorized as male-connoted. Interestingly, Stafff’s tool rates more than 20% of texts as neutral, while this category covers between 2% and almost 10% of texts for the other three providers.

**Table 3:** Percentage of texts assigned to one of the three categories ‘female-connoted’, ‘male-connoted’ and ‘neutral’ by each of the tools. There are two rows per category: the first row represents all data (*n* = 160, 246) while the second row represents data without Google Jobs (*n* = 86, 132).

in %	FührMINT	BetterAds	100W	Stafff
**female-connoted**	72.13	13.47	15.65	-
	73.70	11.74	16.23	33.89
**male-connoted**	17.26	78.72	82.11	-
	16.85	82.26	81.58	43.94
**neutral**	10.62	7.81	2.23	-
	9.45	6.00	2.19	22.17

**Fig 6:**
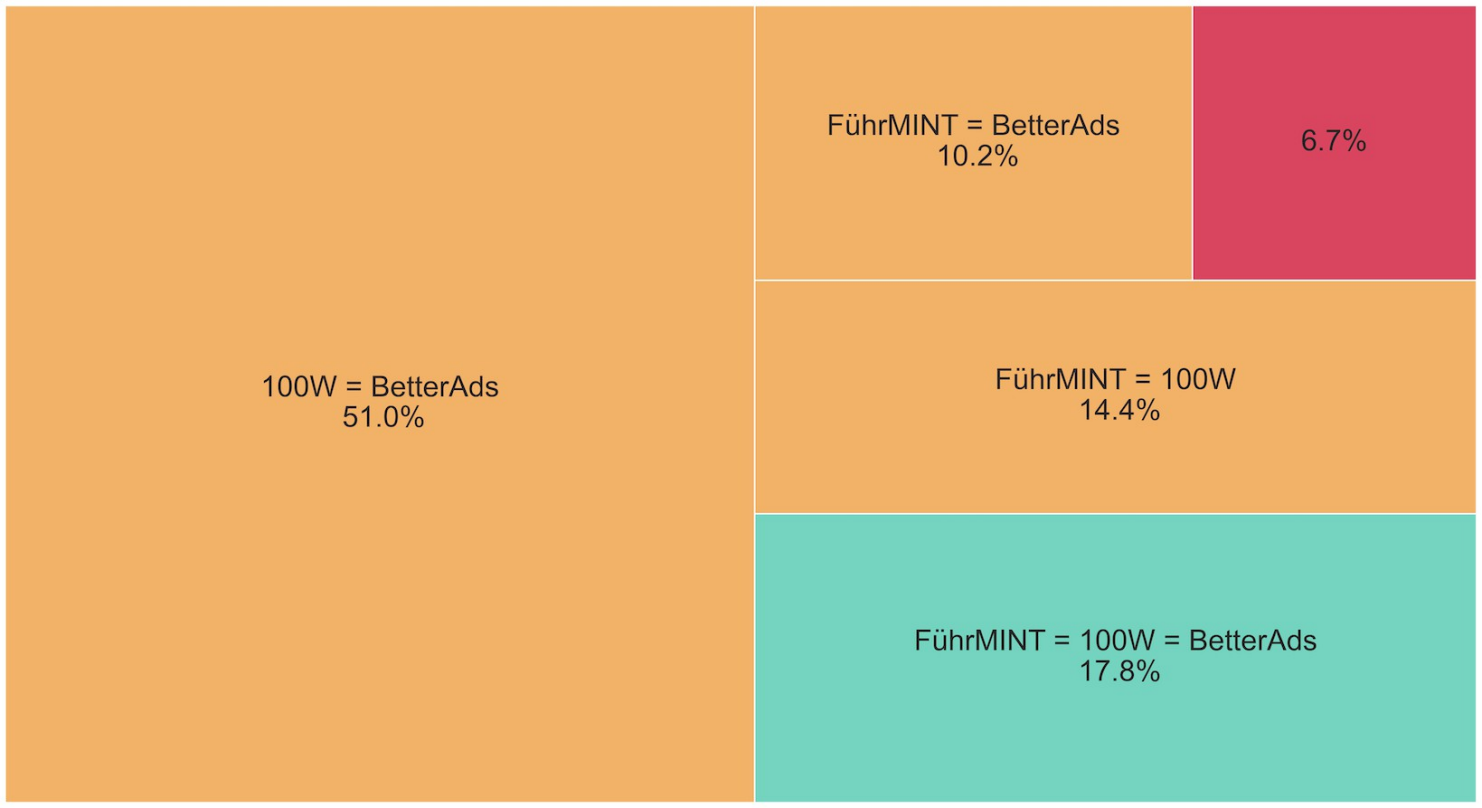
Proportion of texts in the large sample for which none (red), two (orange) or all three (green) of the FührMINT, BetterAds and 100W technologies match.

The differences are also manifested when comparing the assigned categories per text. The categories of all four technologies match in only 11.6% of all cases. At least three out of four tools arrive at the same categorical assessment in 55.3% of them. When providers other than Stafff are compared on the entire corpus, the categories of all three match for almost 18% of the texts, indicating major differences in the assessment processes. Details for the comparison on the entire corpus can be seen in Fig 6.

**Fig 7:**
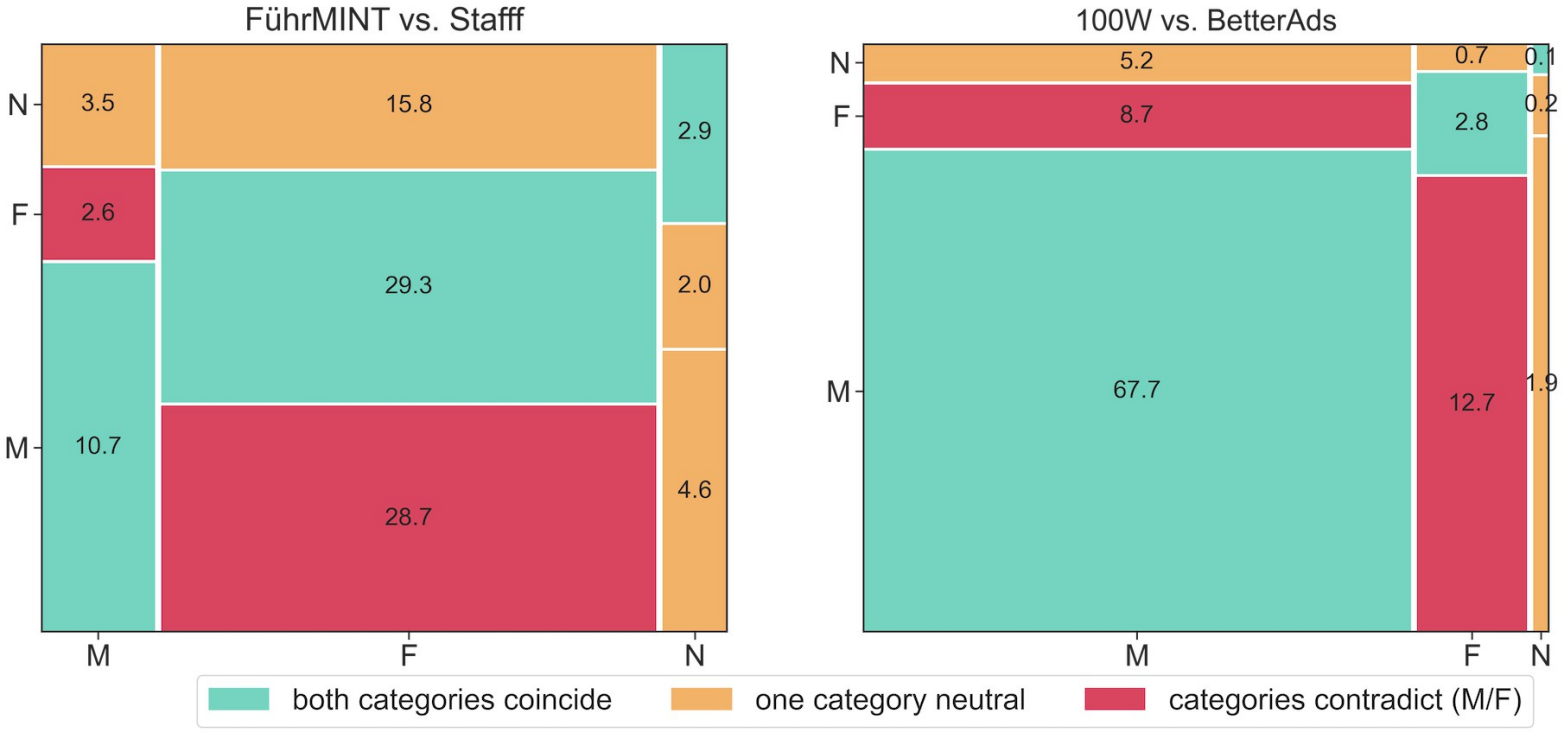
Comparison of FührMINT with Stafff and BetterAds with 100W on the smaller sample. Green indicates matches of both tools (e.g. both female-connoted or both neutral); a box is colored orange if only one of the tools predicts the category neutral; red means that the two technologies arrived at opposite categories. The numbers represent the percentages of texts in the respective groups.

As the distributions of the categories suggest, the two commercial vendors agree more often on their assessments. Fig 7 shows assignment to the same category for over 70% of the texts, while the two providers of freely available software, both based on rather small vocabularies and simple text processing and matching procedures, agree on less than 43%.

### Gender scores

FührMINT Gender Decoder is the only tool that does not compute a score. To allow for comparison with the other products beyond categories, we calculated a score for FührMINT as the difference between the extracted communal and agentic expressions divided by the sum of all extracted expressions. This is a rather natural calculation choice since the category is derived from whether more male-connoted or more female-connoted expressions are extracted.

**Fig 8:**
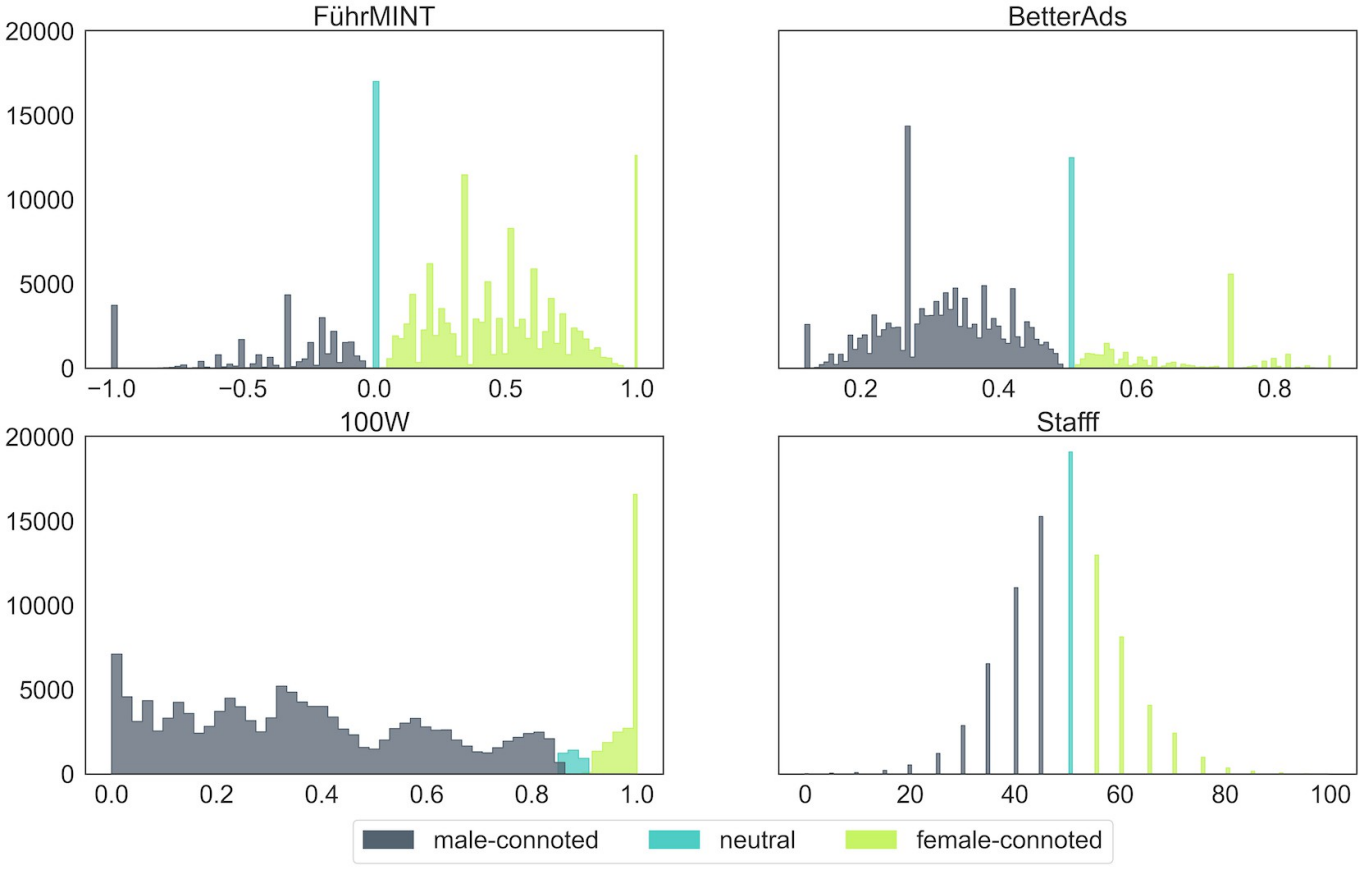
Frequency distribution of gender scores. The colors show the ranges associated with the respective gender categories.

Fig 8 shows the distribution of the scores and their relationship to the categories. Considerable differences in the distributions are immediately apparent. Additionally, 100W stands out in that it classifies a text as neutral if its score is between 0.85 and 0.9, unlike the other products, which use score 0.5. Texts in which the technology found neither male- nor female-connoted words are classified as male-connoted only by 100W; all other three evaluate such a text as neutral.

Equally noticeable are the peaks of 100W and FührMINT at the highest possible score, corresponding to more than 12,000 texts each. However, the overlap between these two text groups is not as high as can be seen from the average text lengths: Those rated by FührMINT as 100% female-connoted are shorter than average, those by 100W are longer. It should be noted that texts scored 100% female-connoted by 100W might contain words or phrases categorized as male-connoted, in contrast to FührMINT. Also remarkable is the fact that the other tools agreed only rarely even on the overall category for these texts, again indicating substantial differences in the overall approaches.

The score ranges as well as the thresholds used to categorize job descriptions differ from tool to tool, which makes the scores themselves not directly comparable. For example, a score of 0.7 for BetterAds would refer to a strongly female-connoted text, while for 100W it would indicate a male-connoted text. We thus conducted the following experiment, which enabled us to compare scoring tendencies. From the texts evaluated by all four technologies (*n* = 86, 132), we drew a sample of 1,000 pairs of distinct job ads and recorded the score tendency for each tool and sample pair, meaning whether the score of the first job ad was higher, lower or equal to the score of the second one. (Note that a pair always consists of two distinct job ads, while a single job ad can occur in multiple pairs.) We then evaluated for how many such pairs the tools agreed on the score tendency. To ensure robustness, we repeated the experiment 10 times.

Interestingly, 100W and FührMINT show a relatively high agreement in score tendencies compared to their assesment of categories. They rate on average 63% of all text pairs in the same direction, and thus agree more often than 100W and BetterAds (58%). The lowest agreement of 50% is achieved between Stafff and BetterAds. In a comparison of all four technologies on the smaller dataset we obtain a full agreement for ∼26% of all pairs. Thus, the technologies agreed more than twice as often when comparing pairs of texts than when comparing categories per text.

**Fig 9:**
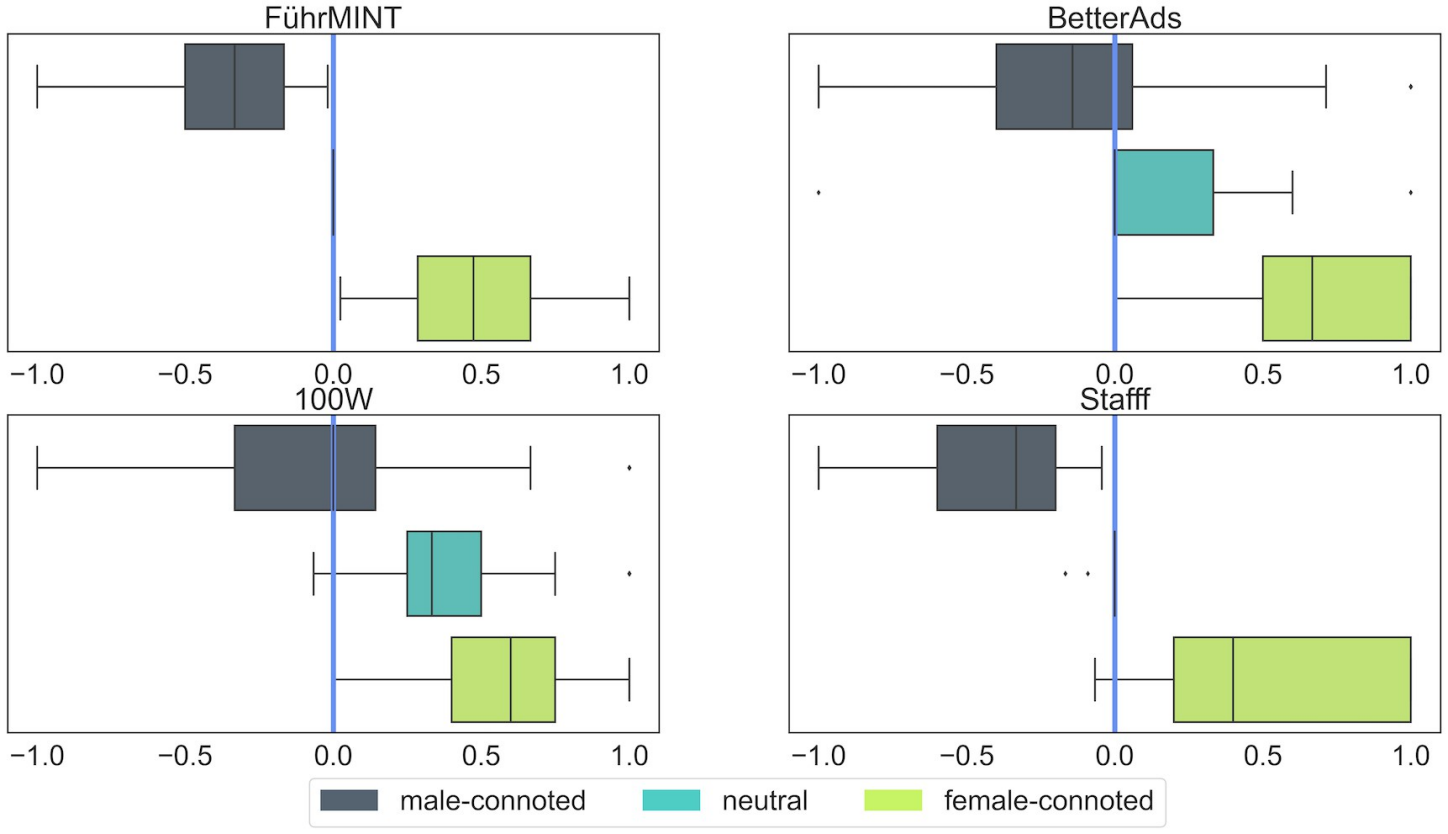
Center and spread of simplified gender scores. The vertical lines refer to the simplified threshold between the gender categories, equal for all four technologies.

To better understand the effect of the actual formulae used to compute the scores and the threshold for the respective gender categories, we calculated a simplified score for each software, using the same formula as for FührMINT: the simplified score equals the difference of the female and male connoted expressions, divided by the sum of both, and a text is evaluated as neutral if the score is 0, i.e. the same number of female and male connoted terms was extracted. In this way, we can directly compare the proportions of the respective extracted words per group between the tools. Fig 9 shows the distribution of simplified scores as boxplots per gender category and software. With the simplified score, the proportion of texts classified as female-connoted by 100W and BetterAds would be significantly higher. With 100W, all texts classified as neutral and half of those classified as male-connoted would fall into the female-connoted category; with BetterAds, we see a similar but somewhat less pronounced effect.

The analyses in this section demonstrate the significance of the chosen formula and threshold. At the same time, it raises the question of how much overlap there is between the extracted words per category, which we will examine in more detail in the following section.

### Matched words and phrases

Significant differences between the technologies are evident at the level of extracted words and phrases. FührMINT only extracts single words due to the simple tokenization and matching procedure, while the other providers also extract short phrases. 100W also extracts longer phrases such as ‘so angenehm wie möglich’ (‘as pleasant as possible’) or ‘ausgeprägtes analytisches Denkvermögen’ (‘strong analytical thinking skills’). Most of the extracted words are verbs, nouns or adjectives; functional words occur only as parts of phrases.

**Fig 10:**
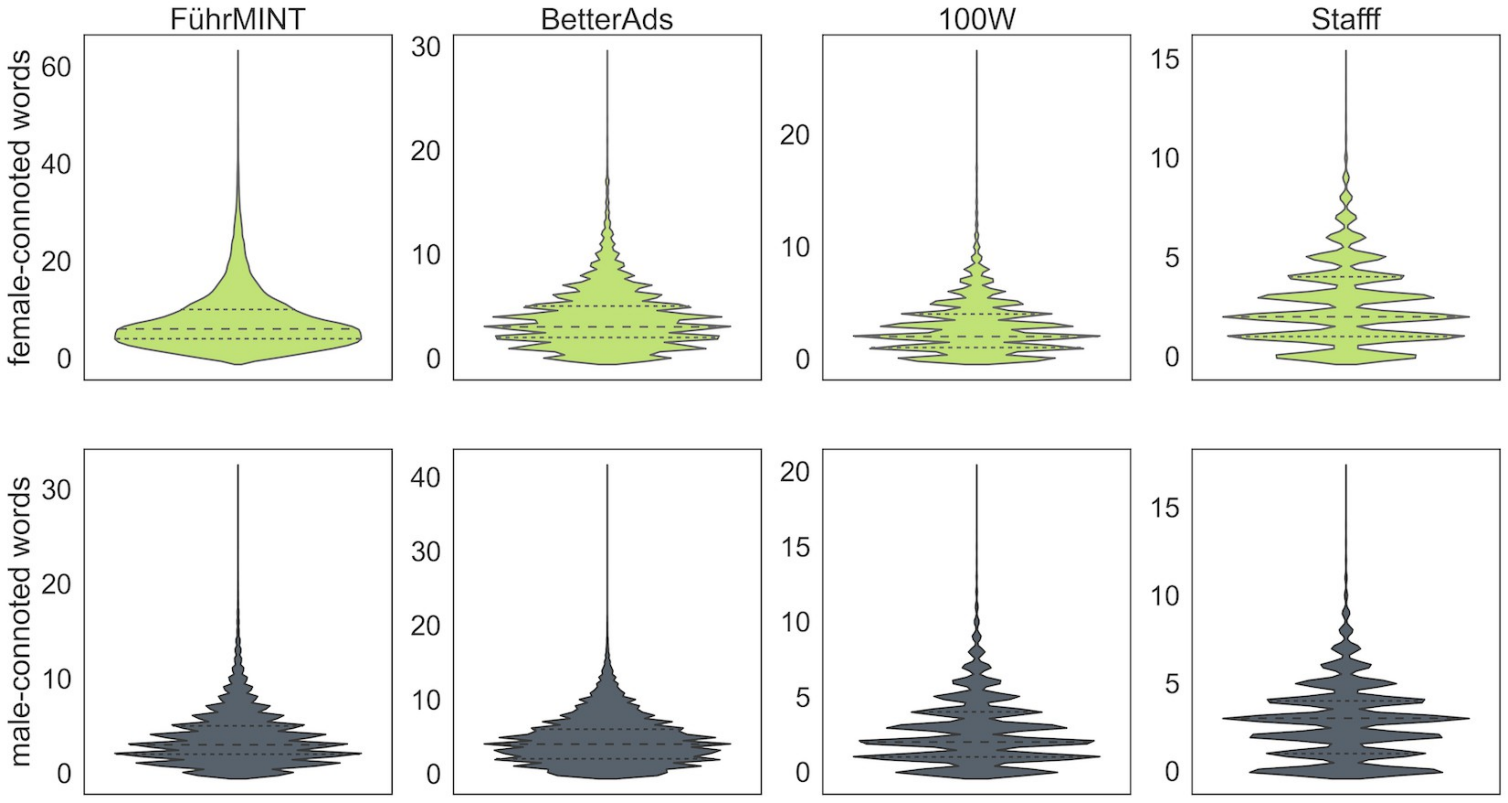
Distribution of the number of extracted feminine and masculine expressions per tool, showing the quartiles as dashed horizontal lines.

The proportion of words or phrases marked as female- or male-connoted by the respective technologies ranges from 1% to ∼3%. The relatively low proportion of gender-connoted terms can be explained by the fact that the full job ads were analyzed, including text zones that contain little information about personality traits such as company description or information about the application process. On average, FührMINT extracts the most female-connoted words per text (>7), which is twice as many as BetterAds and nearly three times as many as 100W and Stafff. For the male-connoted matches, the numbers are much closer. Table 4 displays the average and standard deviation per technology and category, for both datasets. It also shows that examining with and without the Google Jobs corpus does not cause a notable difference at this level. Fig 10 provides a more detailed description of the distribution.

**Table 4:** Average number and standard deviation of matched expressions, split by category (female-/male-connoted), technology and data sample (first and second row representing the large and small data sample, respectively).

average number / standard deviation of matches per text	FührMINT	BetterAds	100W	Stafff
**female-connoted**	7.43 / 6.12	3.77 / 2.96	2.69 / 2.37	-
	7.59 / 6.15	3.87 / 2.89	2.87 / 2.34	2.49 / 1.95
**male-connoted**	3.66 / 2.98	4.19 / 3.15	2.63 / 2.30	-
	3.65 / 2.82	4.58 / 3.23	2.63 / 2.13	2.73 / 2.07

The effect of FührMINT’s very flexible and rather little controlled matching procedure is particularly well demonstrated by the count of unique matches. In the smaller sample, FührMINT finds over 14,000 unique female- or male-connoted words, several times more than the other technologies. In the large sample, which contains about twice as many texts, FührMINT finds about 26,000 unique words, almost twice as many. In contrast, the figure for 100W increases by only 17%. Table 5 contains the 95th percentiles of the corresponding frequency distributions, i.e. the number of the most frequent unique words whose occurrences cover 95% of all matches. These show that a considerable proportion of unique matches is rather rare: FührMINT covers 95% of all extracted words with only ∼2, 000 unique tokens. The 95th percentiles for BetterAds and 100W are very close, and Stafff extracts by far the fewest unique words.

**Table 5:** Total number and 95th percentile of unique matches per technology and data sample. Second row represents smaller data sample. We consider words falling under 95th percentile in order to exclude rare and misspelled words.

unique matches	FührMINT	BetterAds	100W	Stafff
**total**	25,858	8,776	2,249	-
	14,265	5,240	1,921	2,464
**95th percentile**	2,119	403	428	-
	1,537	325	367	200
**matches classified both as female- and male-connoted**	556	52	0	7

The high number of unique matches by FührMINT is caused to a considerable extent by misspelled words, which, in contrast, are not extracted at all by 100W. This indicates that 100W has significantly stronger restrictions regarding the possible matches and in particular does not allow for arbitrary completions of word fragments. This fits well with the fact that there are no words classified both as female-connoted and male-connoted by 100W, in contrast to 556 unique such words for FührMINT. (BetterAds classifies 52 expressions as both; however, it has implemented a mechanism that shows a clear assignment to the user.) Such expressions typically arise as compounds of two or more different words that are associated with distinct categories such as ‘Kooperations*fähigkeit*’ (‘cooperation *skills*’) for BetterAds or ‘Team*führung*’ (‘team *leadership*’) and ‘Fachpflege*kraft*’ (‘specialist *nurse*’) for FührMINT.

**Fig 11:**
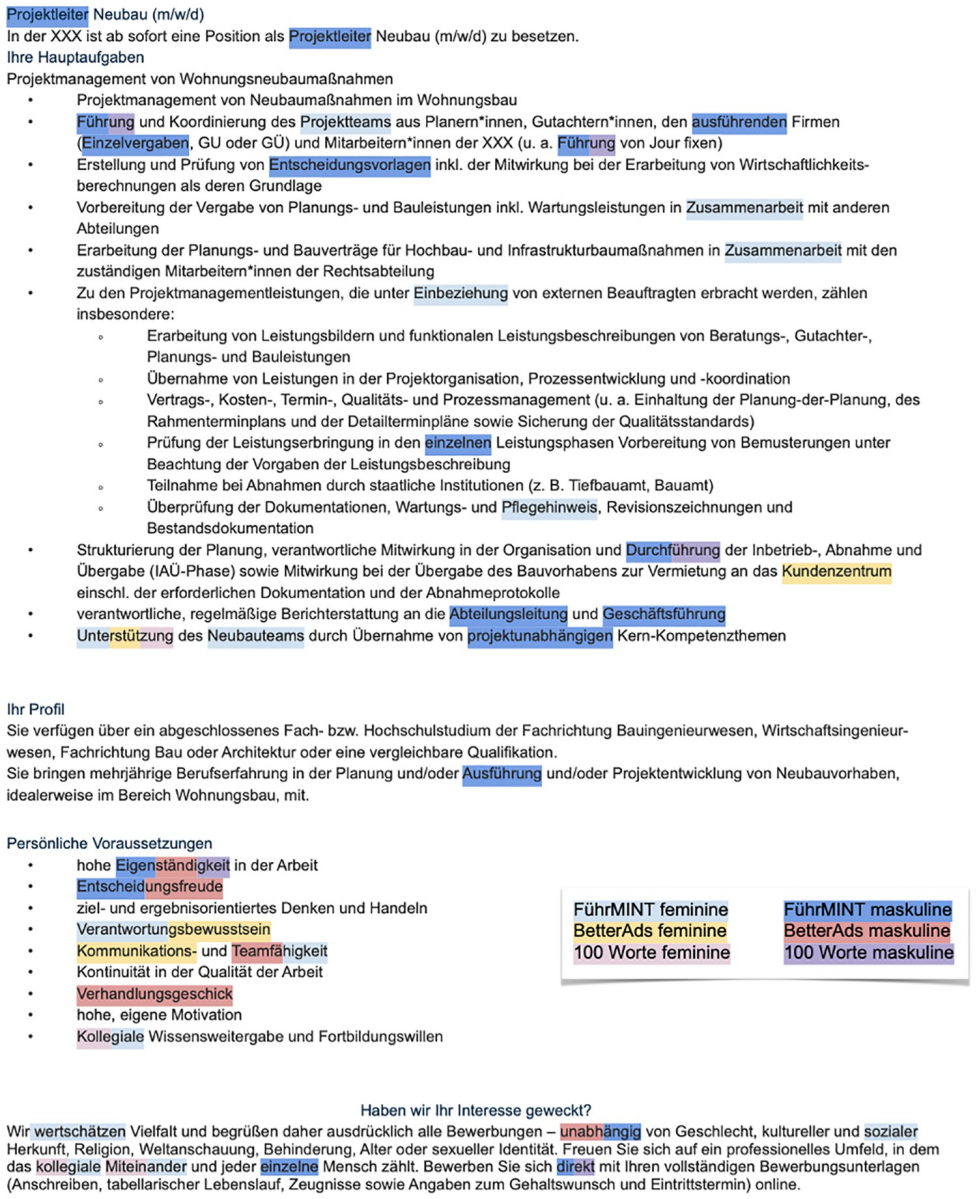
A job posting that serves as an example of how little overlap there is between the three technologies FührMINT, BetterAds and 100W. Only the words ‘Unterstützung’ (‘support’) and ‘Eigenständigkeit’ (‘independence’) are classified by all three technologies in the same way.

Fig 11 shows an example job advertisement with the extracted female and male connoted words for three of the tools. The example illustrates that the two professional, commercial technologies BetterAds and 100W focus in particular on the description of required skills and desired personality traits, while FührMINT also extracts words in other parts of the text, many of which seem unrelated to gender stereotypes (e.g. ‘Einzelvergaben’, meaning ‘individual contracts’, and ‘ausführende’, meaning ‘executive’ but in reference to a company). Moreover, the example indicates that the overall agreement is rather low. For a detailed comparison of the agreement on the level of extracted expressions, we normalized them using the HanTa lemmatizer which showed the best performance on a test sample compared to other state-of-the-art lemmatization methods. HanTa (and other lemmatizers) normalizes expressions by e.g. reducing the plural form of a noun to its singular form or transforming a conjugated verb to its infinitive form. The normalized expressions were subsequently checked and cleaned manually (e.g. combining the frequencies of old and new spellings of the same word such as ‘selbständig’ and ‘selbstständig’, both meaning ‘independent’). The following examinations refer to the normalized word forms.

For each of the four technologies, we have created a list of female- and male-connoted expressions to asses the most frequent expressions per tool. The top five terms already provide a good insight into the vocabularies since they correspond to more than a quarter of all extracted occurrences per tool and gender category. This might not be so surprising considering the level of standardization in formulations of required skills and personality traits in job ads. As shown in Table 6, the top five expressions per gender category indicate a certain level of agreement between the tools, especially for the terms with a female connotation. The latter represent essentially seven different concepts: reliability, support, team and teamwork skills, communication skills, care, customer and flexibility. Four of them occur multiple times, the word ‘zuverlässigkeit’ (‘reliability’) is even present for all four technologies. On the other hand, some expressions are technology-specific: ‘kunde’ (‘customer’) as a stand-alone word and ‘betreuung’ (‘care’) appear each in only one vocabulary; the word ‘flexibilität’ (‘flexibility’), the second most frequently used word with a female connotation in Stafff, only occurs in BetterAds’ vocabulary, but as a word with a male connotation. Another opposing assessment at the word level is given by the term ‘teamfähigkeit’ (‘team capability’) which is among the most frequent five male-connoted expressions for BetterAds, while it belongs to the most frequent five female-connoted ones for FührMINT and 100W. Numerous other examples of opposite associated words can be found when looking at full vocabularies. ‘Selbstvertrauen’ (‘self-confidence’) for instance is categorized by FührMINT as female-connoted, 100W and BetterAds consider it male-connoted. The comparison of the full vocabularies yields 40 unique normalized terms assigned by all four providers to the same gender category, the majority of which being adjectives. Table 7 lists their normalized forms. The vast majority of all extracted terms, however, is specific to only one technology.

**Table 6:** Five most frequently extracted female-connoted and male-connoted words per technology. The word ‘zuverlässigkeit’ (‘reliability’), highlighted in cyan, is the only word occurring in the same category for all four providers, while the word ‘teamfähigkeit’ (‘teamwork’), highlighted in orange, occurs in both categories.

FührMINT	BetterAds	100W	Stafff
**top 5 female-connoted words**			
team (team)	kunde (customer)	zuverlässigkeit (reliability)	zuverlässigkeit (reliability)
betreuung (care)	zuverlässigkeit (reliability)	teamfähigkeit (teamwork skills)	flexibilität (flexibility)
zuverlässigkeit (reliability)	unterstützen (to support)	unterstützung (support)	kommunikationsfähigkeit (communication skills)
teamfähigkeit (teamwork skills)	unterstützung (support)	zuverlässig (reliable)	zuverlässig (reliable)
unterstützen (to support)	zuverlässig (reliable)	kommunikationsfähigkeit (communication skills)	unterstützen (to support)
**top 5 male-connoted words**			
selbstständig (independent)	kenntnis (knowledge)	stärke (strength)	stärke (strength)
stärke (strength)	fähigkeit (ability)	technisch (technical)	belastbarkeit (resilience)
direkt (direct)	teamfähigkeit (teamwork skills)	individuell (individual)	leistungsbereitschaft (willingness to perform)
durchführung (implementation)	individuell (individual)	selbstständig (independent)	selbstständig (independent)
führerschein (driver’s license)	flexibilität (flexibility)	durchführung (implementation)	kompetent (competent)

**Table 7:** Adjectives that have been assigned to the same gender category by all four technologies. Word lists for Stafff were created using data collected from German FEA and Ausbildung.de (*n* = 86, 123).

joint female-connoted words	joint male-connoted words	
ehrlich (honest) engagiert (committed) freundlich (friendly) fürsorglich (caring) gemeinschaftlich (collectively) hilfsbereit (helpful) kooperationsbereit/kooperativ (willing to cooperate/cooperative) unterstützend (supportive) verantwortungsvoll (responsible) zuverlässig (reliable)	abenteuerlich/-lustig (adventurous) aggressiv (aggressive) analytisch (analytical) dominant (dominant) durchsetzungsfähig/-stark (assertive) ehrgeizig (ambitious) eigenständig (independent) entschlossen (determined) hartnäckig (persistent) herausfordernd (challenging)	kompetitiv (competitive) mutig (courageous) offensiv (offensive) rational (rational) selbstbewusst/-sicher (self-confident/self-assured) stark (strong) unabhängig (independent) willensstark (strong-willed)

**Fig 12:**
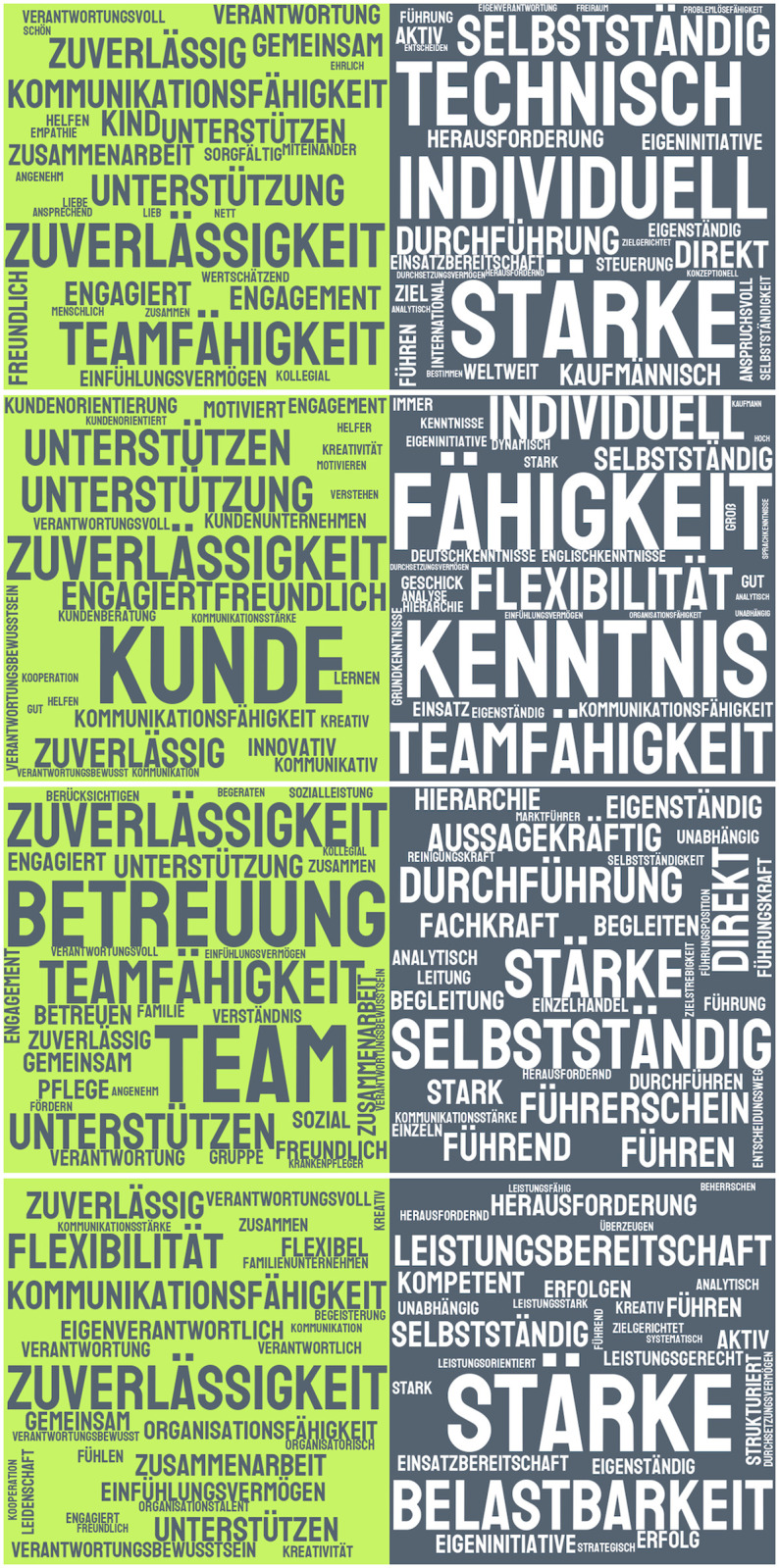
Word clouds of the 30 most frequently extracted words with female and male connotations, after lemmatization. Tools from top to bottom: 100W, BetterAds, FührMINT, Stafff.

It is also worth noting that the number of matches is not necessarily the same for different tools: for example, Stafff finds the word ‘Zuverlässigkeit’ (‘reliability’) ∼34, 000 times, while 100W extracts ∼51, 000 corresponding matches. This shows that the surroundings of the words plays a role; however, Stafff’s regex-based method can be assumed not to take into account any systematic contextual properties of the language. To illustrate the relevance of the most common terms for each technology, we created word clouds per tool and gender category in Fig 12. A comparison of the word clouds in the left column of female-connoted words, for example, clearly shows the most important similarities as well as differences. ‘Support’ and ‘reliability’ have a similarly strong meaning for all four technologies; the word ‘team’ is only relevant for FührMINT; BetterAds focuses on the ‘customer’ (‘Kunde’).

### Segregations in the labor market

Now that we have compared the technologies at the level of individual texts, we turn to the question of how the technologies view groups of texts that reflect different segments of the labor market. Although gender roles and hierarchies have changed over time, gender disparities still manifest in the labor market [68, 69], yielding a relatively stable overall level of gender segregation. Whereas e.g. care work is performed predominantly by women, the technological sector shows an overrepresentation of men among the workforce [70]. The gender-based division can also be observed at hierarchical levels, as women are still underrepresented in leading positions [71].

First, we utilized the job category attribute in the Google Jobs subcorpus to group the texts accordingly. We manually checked samples of the categories for plausibility; except for ‘art, fashion, design’, to which also jo titles such as UX/UI designer were partly allocated, the assignments were considered fitting. We then calculated the proportions per technology, gender category and job category and applied a two proportions Z-test (pooled Z-statistic, 0.95 confidence level) to pairwise compare the figures within each technology and gender category. Cohen’s H was used as a measure of effect size of the calculated proportion differences. Table 8 shows the associated contingency table, in which for each tool and gender category, the job categories with the highest figures are highlighted. It should be noted that statistically, several job categories may be considered as having the highest share, if their respective proportions do not differ in a statistically significant way, such as ‘real estate’ and ‘science and engineering’ for male-connoted jobs and 100W.

**Table 8:** Proportions of job ads per job category classified as male-connoted (m), female-connoted (f), or neutral (n), using Google Jobs as the underlying data. The color-highlighted cells correspond to job categories with the statistically significant highest proportions per tool and gender category (green for female-connoted and grey for male-connoted).

job category	count	FührMINT			BetterAds			100W		
		m	f	n	m	f	n	m	f	n
construction	5,539	31.5	53.8	14.6	73.9	15.0	11.1	90.3	7.7	2.1
education	5,576	9.8	78.9	11.3	61.3	21.4	17.3	66.3	31.2	2.6
accounting and finance	5,723	19.7	69.4	10.9	82.6	10.4	7.0	88.6	9.4	2.1
computers and IT	4,026	15.2	74.8	10.0	86.1	8.4	5.5	87.6	10.0	2.4
gastronomy	5,763	8.7	76.4	14.8	63.4	21.3	15.2	76.2	21.3	2.5
health, care and social services	6,632	4.3	90.5	5.2	65.3	21.4	13.4	64.2	32.1	3.6
real estate	4,557	19.0	66.4	14.6	84.8	8.8	6.3	91.4	7.5	1.1
installation, maintenance and repair	5,864	23.8	62.3	13.9	78.8	13.2	8.1	90.2	7.9	1.9
art, fashion and design	4,253	15.8	71.1	13.2	72.7	19.0	8.3	85.0	12.5	2.5
management	4,149	28.9	60.6	10.4	80.1	12.8	7.0	87.8	10.1	2.2
media, communication and writing	4,732	12.4	76.0	11.6	74.5	17.8	7.8	84.2	13.1	2.7
cleaning and facility services	5,075	14.5	73.6	11.9	62.9	22.6	14.5	79.3	18.9	1.9
security professions	4,770	29.2	56.8	14.0	81.2	11.0	7.8	89.1	9.0	1.9
science and engineering	4,205	26.0	60.7	13.3	87.0	7.2	5.9	91.5	6.7	1.8
total	70,864	18.0	70.0	12.0	74.6	15.4	10.0	82.9	14.8	2.3

The comparison of the proportions shows mainly two things: for one, the technologies have many similarities in the ranking of the respective occupational categories. More precisely, the figures per job category and gender category mostly point in the same direction from the overall assessment which is displayed in the last row of Table 8. For another, the trends are broadly consistent with labor market segregation. The category ‘health, care, social services’ has the highest proportions of female-connoted job ads for all three technologies. In ‘education’, 100W and BetterAds also have the highest proportion of female-connoted texts; FührMINT shows the same tendency. For BetterAds there are two other categories in the same range, namely ‘gastronomy’ and ‘cleaning and building services’. The latter, however, does not stand out for the other two providers.

There is no job category that has the highest percentage of male-connoted texts at all three providers. ‘Construction’ stands out at FührMINT and 100W; ‘science and engineering’ at 100W and BetterAds; ‘real estate’ is also among the categories with the highest proportions of male-connoted texts for 100W, and BetterAds shows the same tendency, while the figures for FührMINT are rather close to average. ‘Computers and IT’ stands out as the only job category with noticeable contradictions: in BetterAds it has the highest percentage of male-connoted job ads, while under FührMINT it has the opposite tendency; in 100W it is rated relatively average.

For vertical segregation in the labor market, the description of managerial jobs is particularly interesting. In [8], referred to by all four providers, the root word ‘lead’ is listed as an agentic term. In German, it can be translated primarily as ‘führen’ and ‘leiten’. Both stems ‘führ’ and ‘leit’ are listed in the FührMINT dictionary of male-connoted word fragments, and thus all words containing these strings are classified by FührMINT accordingly. This example shows again why simple substring completion without further restrictions can be problematic, as words like ‘Gleitzeit’ (‘flextime’) oder ‘Bleitöpfe’ (‘lead pots’) also contain the word ‘leit’ without having any semantic similarity with it (in other words, ‘leit’ is not a meaning-bearing morpheme for these words). 100W considers ‘führen’ and ‘leiten’ as male-connoted words, too. They extract them as verbs in all conjugations and tenses and partly as nouns (‘leadership’) but they do not extract compound words, such as ‘Führungskompetenz’ (‘leadership competence’). Interestingly, BetterAds does not seem to include the two verbs in either group. The words extracted by BetterAds that included ‘führ’ or ‘leit’ seem to have been found through other word parts, e.g., ‘Führungsfähigkeit’ (‘leadership’) as a male-connoted word because of the suffix ‘fähigkeit’ (‘ability’) and ‘Kommunikationsführung’ (‘communication leadership’) as a female-connoted word due to the prefix ‘Kommunikation’ (‘communication’). For Stafff it was not possible for us to recognize a pattern in the matches.

To allow for a quantitative comparison, we created two independent sets of job ads based on keywords typically describing leading positions. In the ‘positive sample’, we included all job ads for which the job title contained clearly manegerial words. The ‘negative sample’ comprised those job postings that contain no terms that could be related to a managerial position in either the title or the job description. The two samples were checked for accuracy on a random subsample. We used the remainder of the data as a third data sample ‘unknown’. The descried procedure yielded 32,5% (*n* = 52, 100) in the negative and 3% (*n* = 4, 789) in the positive sample.

**Fig 13:**
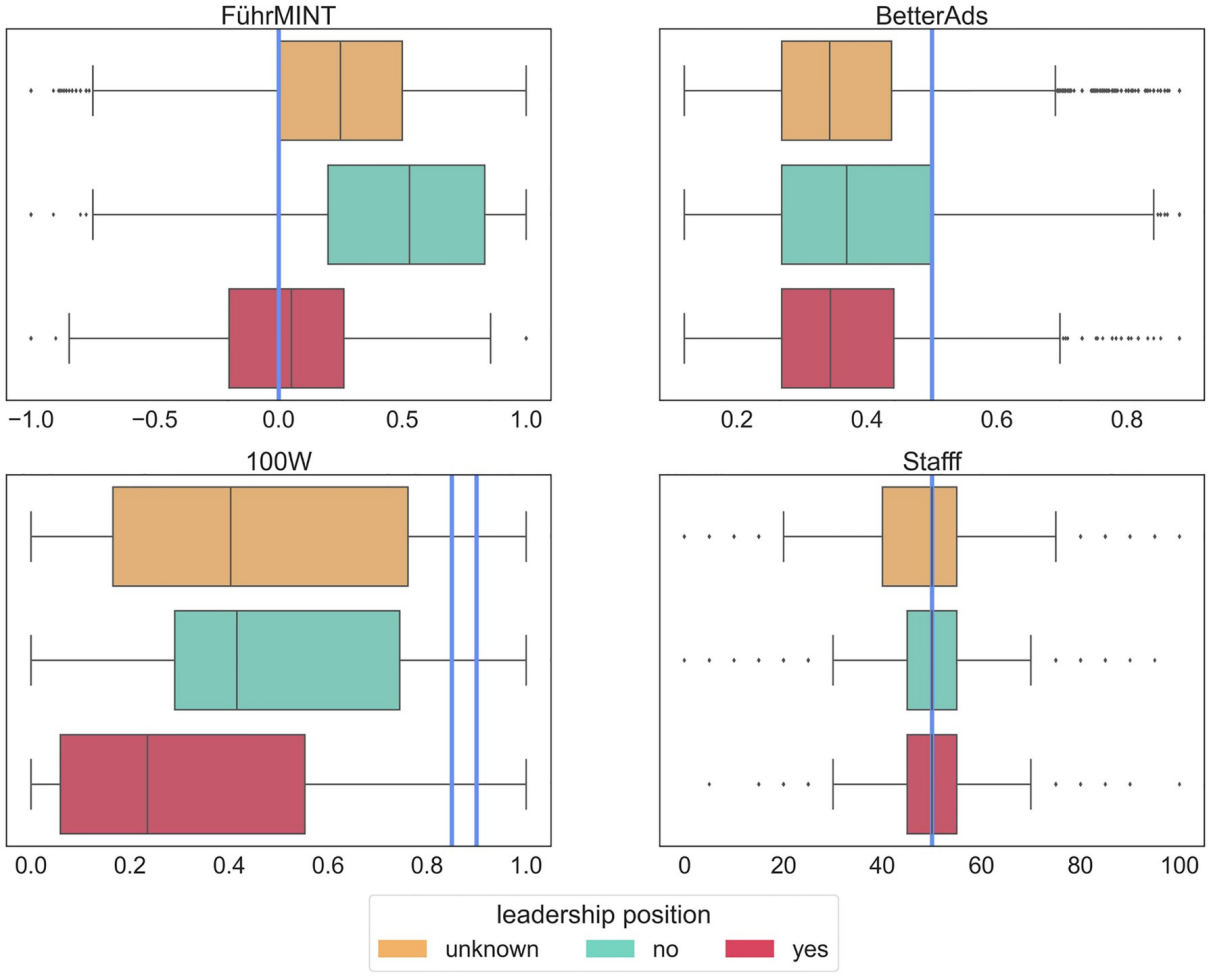
Box plots of the distribution of gender scores in three data samples: Leadership jobs, jobs without leadership requirements, and jobs for which leadership information is not known.

As for job categories we performed a two proportions Z-test (pooled Z-statistic, 0.95 confidence level) to pairwise compare the figures within each technology and gender category. As can be seen in Table 9, all tools except for Stafff show a significantly higher proportion of male-connoted texts in the positive sample of leadership jobs and are thus aligned with vertical segregation. The difference is most pronounced for FührMINT when comparing the positive and the negative sample (Cohen’s H = 0.83). For 100W the effect sizes are rather small (max h = 0.18), similar as for BetterAds (max h = 0.29). Interestingly, 100W and BetterAds are similar in their respective ratings of male-connoted job ads, although BetterAds does not explicitly rank words related to leadership as male-connoted. This indicates that leadership positions have masculine connotations via words other than those derived from the verb ‘lead’. For a detailed comparison, we also visualized the scores in the respective samples as box plots in Fig 13.

**Table 9:** Percentage of job ads classified as having a male connotation (m), a female connotation (f), or a neutral connotation (n), broken down by leadership. The color-highlighted cells correspond to the groups of job ads (leadership: yes/no/unknown) with the statistically significant highest proportions per tool and gender category (green for female-connoted and grey for male-connoted).

leadership position	count	FührMINT			BetterAds			100W			Stafff		
		m	f	n	m	f	n	m	f	n	m	f	n
yes	4,178	40.7	51.6	7.7	82.1	11.5	6.4	87.6	10.8	1.6	43.6	36.5	19.9
no	52,100	7.5	78.9	13.5	70.6	16.1	13.3	83.8	14.9	1.3	42.8	30.9	26.3
unknown	103,357	21.1	69.6	9.3	82.7	12.2	5.1	81.0	16.3	2.7	44.6	35.6	19.8
total	160,246	17.3	72.1	10.6	78.7	13.5	7.8	82.1	15.7	2.2	43.9	33.9	22.2

## Summary

We have identified four providers of technologies for screening and correcting gender-based exclusion potentials of job advertisements on the German-speaking market and examined both their theoretical foundations and, as far as accessible to us, the main processing steps of their software. We have done this both qualitatively, based on an extensive literature review as well as on conversations with the software providers and screening of their websites, and quantitatively based on a set of German-language job description texts comprising more than 160,000 samples from three different job platforms.

All four technologies draw on scientific research from the field of social psychology, particularly on agentic and communal traits and their linguistic manifestations. Therefore, they share a common scientific reference that might suggest that job advertisement texts might be similarly classified. However, as we were able to show based on our corpus, this is not the case, answering our first research question (Q1) in the negative. The technologies’ assessments diverged immensely at several levels of comparison: The assignment of a text to one of the three categories yielded low levels of agreement, with e.g. FührMINT assessing more than 70% of job ads predominantly as female-connoted while Beesite BetterAds and 100W consider around 80% to be rather male-connoted. We further tested on random pairs of job ads how often the technologies agreed on whether one text was more or less male-connoted than the other, with the result that all four technologies agreed on only a quarter of the comparisons. Moreover, the overlap of all words identified as female- or male-connoted was relatively small.

While the evaluation of individual texts varied considerably, commonalities could be observed at less granular levels. Although the overall vocabularies show little overlap, there are many common words among the most frequently extracted terms, most of which are adjectives associated with agentic and communal traits. When texts are grouped by their respective job categories, the three technologies compared—FührMINT, BetterAds, and 100W—show similar trends and evaluate, for example, the category ‘health, care, social services’ as one with the most female-connoted texts. These three also rate job ads for leadership positions as male-connoted to a significantly higher degree than those that do not involve a leadership role. It can thus be concluded that at least these three technologies show a common basic tendency and allow similar observations of the horizontal and vertical segmentation of the labor market. In detail, however, all four differ in a significant way, which brings us to our second research question (Q2) of how the technological levers influence the final result and ultimately lead to the respective differences.

The basis of each of the technologies are gender-connoted words which are drawn from existing research and weighted by the vendors them selves. As a consequence, differences arise such as e.g. in deciding whether the verb ‘lead’ should be considered agentic or not. Why a certain research finds its way into the dictionaries or not can have many reasons, scientific as well as strategic. What is certain, however, is that in the case of FührMINT, BetterAds and 100W, experts from social or work psychology were involved in this process, which on the one hand implies expertise and at the same time shows that the human factor plays an important role. The identification of words with specific meaning is not a trivial task, since human language is multifaceted with semantic, cognitive and social aspects. A string-based search for words completing certain word stems, as implemented for example by FührMINT and not too differently by Stafff, yields many false positives that might confuse users of the software. State-of-the-art techniques such as part-of-speech tagging or word embeddings make it possible to perform searches that are able to distinguish different meanings of a word in many settings. In the case of 100W and BetterAds technologies, their application leads to more plausible and consistent results. However, the development and implementation of such algorithms requires expertise in natural language processing and machine learning. Nevertheless, based on our analyses we would recommend providers of respective software to develop and use algorithms that can assess the meaning of an expression if feasible. The application of such algorithms would also be worth considering for scientific software, in psychology and beyond, that relies on occurrences of certain concepts in human language.

With social psychological research and adequate search procedures, queried concepts can be extracted from job ads. The technologies considered implement different and at the same time far-reaching decisions. As we could show, the gender category distribution of 100W and BetterAds would be much more similar to that of FührMINT if all applied the same simplified score (a normalized difference of the number of words in both groups) and the same threshold to assign gender categories. It is not possible to say which score and threshold would be better, because in our opinion there is a lack of relevant research so far.

Our results contribute to research on validity of automated language-based models for personnel assessment. We found that the four technologies produce different results, despite being based on similar and partly overlapping scientific theories and findings. Thus, we have shed light on the practical challenges of real-world scientific implementation by highlighting the underlying mechanisms of bias detection algorithms. We cannot claim that one technological implementation is better than the other. We appreciate the transparency of FührMINT as well as the elaborate preparation of texts at 100W and BetterAds or their generation of suggestions. The usefulness of the technologies must ultimately be tested in real-world scenarios, and in the discussion we offer perspectives and questions for further research.

## Discussion

### Implications for HR practice

Due to the war for talents and the growing demand for diversity as a (competitive) advantage [72], the technologies introduced might be an adequate solution for HR departments to attract a heterogenous pool of applicants. Some companies using such technologies have claimed that they fill jobs more quickly and that the application ratio increases – Johnson & Johnson, for example, stated that the use of Textio, a leading US-based Augmented Writing software, led to a 9% increase of female applications (additional 90,000 applicants) [73].

However, potential users of similar technologies should be aware that the technologization and automation of scientific knowledge in the context of HR software (and beyond) often involves a multitude of decisions on the part of developers that can vary arbitrarily. When evaluating technologies for gender-based exclusion detection, we recommend making sure that at least (1) experts from the relevant scientific field were involved in the development, (2) the ideas behind the research as well as the implementation are made transparent to users, (3) the algorithmic processing is appropriate and purposeful, (4) the limitations of the application are brought to light, and (5) that the vendor has extensively evaluated the technology in settings similar enough to one’s own. Some of the aspects become clear through the use of the technologies and application to examples, which is why we recommend trying out technologies extensively using different samples with different groups of people.

The assessment of the scientific basis itself can generally be difficult for HR practitioners. It is nevertheless important as technologies exist on the HR market that refer to scientific research that is controversial within the respective academic community. Recent tools in the field of ‘emotion analytics’ calculate scores for employability of applicants based on psychological theory about facial expressions and ‘universal emotions’. However, the referenced theory has hardly been confirmed in the real world and is the subject of controversial debate [28, 74]. The importance of evaluations on data that correspond to the actual purpose of a software is demonstrated, for example, by the recent analysis of IBM Watson Personality Insights, a technology for language-based personality assessment that is based on self-reports of Twitter users [26]. It turns out that the tool’s scores show little agreement with self and observer ratings in the context of video interviews, showing the unsuitability of the underlying data source for the actual use case.

When looking out for tools that help remove (gender) biases in job ads, potential users should carefully consider that it is not enough to use a software for diversity-based targeted strategies, but rather that it can aid as a systematic prescreen that, yet, needs to be checked by recruiters with the respective expertise. As suggested by Frost and Alidina [75], such technologies are “a supplement to help hiring managers mitigate their bias, and are not a cure-all”. Another effect to be avoided is automated trust, which could lead to relying entirely on the judgment of technology and perhaps drawing wrong conclusions if the expected effects do not materialize after all, or believing that the issue of diversity can be ticked off. Even if the pool of applicants was to become more diverse, the assumption that everyone now has the same starting conditions, for example, would still be wrong. It must be emphasized that the use of such technologies needs to be coordinated within the organizational structure and reflected as a holistic approach in HR management and structurally anchored within the organizational framework. Otherwise, there is a risk that the topic of diversity and respective responsibilities will be shifted to the technologies.

We are well aware that it is extremely difficult to develop a technology that reduces exclusion. There are multiple reasons for not applying for a job, and these are difficult to figure out in detail even in small case studies with possibilities to gain in-depth and partly unconscious insights. The technological translation can only be limited, and for practitioners, especially those with an interest in improving social conditions, it might be fruitful to take the perspective of “algorithmic realism”, and consider “values they may be taking for granted, […] how to responsibly account for unexpected impacts […] and whether an algorithm actually provides an appropriate intervention” as an integral part of their work [76].

### Opportunities and challenges for gender equality

The evaluated tools aim to contribute to the promotion of gender equality. Their daily use in companies can provide an opportunity to question current gender-based structures and to deal with possible exclusions in the organization. Furthermore, they foster an awareness of the power of language and promote an examination of the possible effects of wording on exclusion in hiring processes and beyond. Bogen and Rieke [27] argue with respect to comparable technologies in the US, “Even if the predictions they offer are imperfect, such tools still prompt employers to spend time trying to make their descriptions more inclusive”.

Components for decoding gender bias could find their way into many HR software solutions and even job portals. Stepstone, for example, one of the most important job portals on the German-speaking market, recently added such an open access component to its portfolio [77]. Here, too, reference is made to the study by Gaucher et al. [8]. Provided that the trend continues and corresponding technologies are deployed on a larger market scale, their usage could have even further positive effects. As a bottom line to similar providers in the US market, Bogen and Rieke [27] state that “since a number of other predictive hiring products—from job ads to screening tools— rely on the words and phrases from job descriptions to inform their predictions about candidates’ suitability, more inclusive language in job postings can influence everything from who ends up seeing job ads to who is invited to interview”.

However, in order to counteract the dichotomous way of approaching gender, we consider it advisable to promote ‘gender neutrality’ as far as possible. This is not, or not consistently, reflected in the technologies studied, though. All technologies show male- and female-connoted words, partly highlighted in blue and pink; neutral descriptions of personality traits are not explicitly presented. Yet, neutral words are not provided by existing research; at least, we are not aware of any research studies that explicitly declare and test gender-neutral words, so providing such terms is currently unlikely to be feasible. It should be noted, though, that some tools provide suggestions for substituting male-connoted words. The suggested words are presumably those with a known female connotation or those for which no gender connotation has yet been demonstrated in research. However, words that have not been show to be female- or male-connoted by existing research can still have a gender-related effect. In the Appcast study [78], roughly speaking, texts without words from Gaucher et al. [8] were designated as texts written in gender neutral language and their click and application rates were evaluated. Accordingly, caution should be used in interpreting the results for such texts.

In addition, it is important to note that the technologies under consideration could perpetuate attribution processes among users, especially recruiters. Particularly with regard to words related to leadership, it may result in an unconscious manifestation of the stereotype that women are not suitable for leadership roles and thus reinforce vertical segregation or contribute to the development of new biases. In this context, it would be interesting to examine whether the use of terms such as ‘push’ and ‘pull’ (BetterAds), which have no explicit reference to gender, would be more sensible. In addition, more research on targeted recruitment strategies for stigmatized identities would be interesting [17]; specifically in labelling neutral job titles and requirements for attracting (female) jobseekers and other underrepresented groups in the labour market.

### Future research

A large number of scientific studies have demonstrated under laboratory conditions that the choice of words in advertisement texts can have an influence on whether a woman wants to apply for the corresponding position. The technologies under investigation build on parts of this research [8, 16, 21]. However, they make a number of choices during software development and arrive at some widely varying judgments of individual ads. This emphasizes the need for validation studies that examine the real-world implications of using such technologies. Some providers advertise their products with numbers for increased success; BetterAds even reports on an A/B test based on 50 job ads from three different employers. Such testimonials may be an indication of positive real-world effects. However, (further) respective scientific studies are not yet available, and only those will make it possible to assess the benefits of these technologies in detecting presumed bias in job postings.

Moreover, the settings in which the relevant studies from social psychology were conducted raise the question of the extent to which this effect would also be positive under an intersectional perspective. At this point, it should be noted that the theories themselves were mostly developed in studies with white college students; this begs the question whether the technologies would also benefit primarily white women with better socioeconomic backgrounds (cf. e.g. [79]).

The social psychological results mentioned are not free of contradictions [9]; research shows that, for example, agentic/communal (self-) perception experiences a partial shift [14, 80]. This demonstrates a need for replication of studies such as [8] and partly explains why technologies reach different conclusions as shown in our empirical analysis, in that company experts might interpret literature differently. Research along the lines of [9] towards the development of standardized gender-specific word lists that can serve as a reliable basis for computational text analyses would certainly put appropriate technologies on a more stable footing.

Scores and gender categories derived from underlying word lists generally improve when a text contains more female-connoted words: for three out of four providers, a text is classified as (more or less) neutral if the number of terms from both categories is roughly balanced. However, we are not aware of any research that quantifies the complex interaction of words and claims that female-connoted words can ‘neutralize’ male-connoted ones. Social psychological research could certainly provide insights and, for example, examine the effect of repeating a single male-connoted word multiple times. It could also make sense for developers of respective technologies to simply dispense with scores altogether or at least to use very simple formulas that are fully understandable and transparent to users. Further studies in the area of human computer interaction could be fruitful in this regard.

Future research could also examine how HR departments work with the software to capture the implications for structural changes; especially, as to find out whether cognitive effects such as moral credential and automated trust will lead practitioners to rely too strictly on the outcome. However, it should be noted that such studies are not easy to implement since licenses need to be provided, and typically the companies do not only give access to the tools but also provide extensive resources on how to use them. In addition, there is also the difficulty of data protection: there are hardly any established procedures so far on how to actually carry out bias-assessment of technologies, since companies are not supposed to store sensitive personal data such as gender and origin due to the General Data Protection Regulation (GDPR) but also the General Equal Treatment Act (AGG). Thus, research is also needed on how such analyses can be legally implemented. Besides, it would be valuable to elaborate how an external auditing for developing accountable and transparent technologies could be rolled out as to provide systematic guidance for third-party algorithm auditors and regulators (see e.g. [81]).

An exploratory analysis of the user experience would be fruitful in order to create more reliable technology. For this purpose an interdisciplinary exchange between companies and researchers is needed: While technology implementers, especially for-profit organizations, aim to provide solutions that are economically profitable, researchers studying social biases examine how and whether inequality relationships are reproduced. Both sides and their incentives have to be better aligned by ensuring translation work and exchange on behalf of scientific grounding. Ultimately, both parts should aim to develop reliable technologies to promote (gender) equality due to societal but also economic reasons.

### The question of foundations: Social psychology vs. predictive models

The assessed technologies leave the limited scope of underlying research, which itself is in part affected by measurement bias as it is based mostly on small case studies of homogeneous subject groups, by aiming to analyze arbitrary texts and assess their exclusion effect on arbitrary women. The gap between the scope of studies from social psychology and the breadth to which the software is applied cannot be closed. The approach also seems unlikely to scale, both toward broader linguistic considerations, updates regarding temporal changes, and especially not toward intersectionality.

An interesting alternative could be predictive approaches, as applied e.g. by Textio, that make use of statistical relationships between linguistic expressions and target variables such as the proportion of women among applicants. “In job posts, for example, the words ‘exhaustive’ and ‘fearless’ are statistically more likely to result in more men applying than women—there’s no gender-bias phrase list that would include these. They’re not intuitive as masculine-bias words, but it’s what the current data show” [82]. Textio highlights the possibility that the exclusion potential of individual phrases may change over time and explicitly distances itself from “simplistic ‘bias checker’ software” that they consider to be “based on outdated research, with few controls on data integrity” [83], instead taking a purely data-driven approach [84].

Access to application texts on the one hand and the outcome (number and demographics of applicants) on the other make predictive approaches possible. Provided that other attributes such as age and social background are also available in the outcome data, such methods can be extended to cover additional diversity dimensions. But such approaches suffer from other problems. One can only model what is in the data, and people who do not apply, even though they may be professionally suitable and looking for a similar job, will not be represented. Nevertheless, it would be interesting to conduct our analysis as well as impact studies using social psychology-based tools and those with a predictive data-driven approach to shed more light on the practical potential of the respective technologies.
